# Upconversion FRET quantitation: the role of donor photoexcitation mode and compositional architecture on the decay and intensity based responses

**DOI:** 10.1038/s41377-022-00946-x

**Published:** 2022-08-19

**Authors:** Agata M. Kotulska, Aleksandra Pilch-Wróbel, Satu Lahtinen, Tero Soukka, Artur Bednarkiewicz

**Affiliations:** 1 Division of Biomedical Physicochemistry, Institute of Low Temperature and Structure Research, PAN, ul. Okolna 2, Wrocław, 50-422 Poland; 2 Department of Life Technologies/Biotechnology, University of Turku, Kiinamyllynkatu 10, 20520 Turku, Finland

**Keywords:** Nanoparticles, Biophotonics

## Abstract

Lanthanide-doped colloidal nanoparticles capable of photon upconversion (UC) offer long luminescence lifetimes, narrowband absorption and emission spectra, and efficient anti-Stokes emission. These features are highly advantageous for Förster Resonance Energy Transfer (FRET) based detection. Upconverting nanoparticles (UCNPs) as donors may solve the existing problems of molecular FRET systems, such as photobleaching and limitations in quantitative analysis, but these new labels also bring new challenges. Here we have studied the impact of the core-shell compositional architecture of upconverting nanoparticle donors and the mode of photoexcitation on the performance of UC-FRET from UCNPs to Rose Bengal (RB) molecular acceptor. We have quantitatively compared luminescence rise and decay kinetics of Er^3+^ emission using core-only NaYF_4_: 20% Yb, 2% Er and core-shell NaYF_4_: 20% Yb @ NaYF_4_: 20% Yb, 5% Er donor UCNPs under three photoexcitation schemes: (1) direct short-pulse photoexcitation of Er^3+^ at 520 nm; indirect photoexcitation of Er^3+^ through Yb^3+^ sensitizer with (2) 980 nm short (5–7 ns) or (3) 980 nm long (4 ms) laser pulses. The donor luminescence kinetics and steady-state emission spectra differed between the UCNP architectures and excitation schemes. Aiming for highly sensitive kinetic upconversion FRET-based biomolecular assays, the experimental results underline the complexity of the excitation and energy-migration mechanisms affecting the Er^3+^ donor responses and suggest ways to optimize the photoexcitation scheme and the architecture of the UCNPs used as luminescent donors.

## Abstract (web summary)

Upconverting FRET assays show complex behavior of luminescence kinetics and spectra for various NPs chemical architectures and excitation schemes.

## Graphical abstract

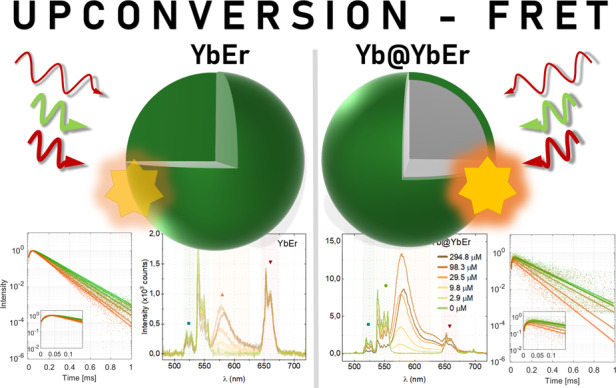

## Introduction

Förster resonance energy transfer is one of the most useful spectroscopic techniques to study biological interactions such as DNA hybridization, antibody-antigen binding, protein folding, and enzyme activity, which have found numerous applications in fundamental biology^1^, medical diagnostics^2,3^, and biotechnology^4^ studies. In general, spectrally overlapping and spatially co-localized energy donor (*D*) and energy acceptor (*A*) molecules enable *D* to non-radiatively donate energy to *A*, which is evidenced by *D* quenching and sensitized *A* emission under selective *D* photostimulation. Concomitantly, *D* excited states lifetimes get shorter for the rising amount of proximal *A* species. Despite numerous successful applications in biomedical diagnostics or functional imaging, several factors affect the reliability of molecular FRET quantification. Quantitative and sensitive FRET-based biosensing is challenging, either in spectral or time domain^5^. This is caused by common artifacts that accompany the measurements^6^—for example, (i) direct *A* excitation under *D* photostimulation, (ii) spectral overlap of *D* and *A* fluorescence leading to difficulties in distinguishing the signal from the *D* and *A* molecules, (iii) typical coexistence of *D* emission with sample autofluorescence, (iv) environmental effects on photophysical properties of *D* or *A* molecules, (v) short ps – ns fluorescence decays of donor molecules, and (vi) photobleaching of *D* and *A* molecules. At first glance, all these issues can be addressed by Upconverting nanoparticles (UCNPs), which eliminate direct acceptor photoexcitation and sample autofluorescence by combining the energy of multiple low-energy photons towards a single high-energy photon in the process known as energy transfer upconversion^7^. Moreover, because the UCNPs are photostable, weakly dependent on the environment, and exhibit long (µs-ms range) luminescence lifetimes, they have been considered attractive alternatives to conventional molecular FRET donors^8–10^ and applied in the measurement of e.g., DNA hybridization^11^, folic acid in the whole blood^12^, or antigen–antibody reactions^13^.

Nevertheless, the upconversion (UC) in UCNPs (e.g., NaYF_4_: Yb, Er) is relatively a complex combination of multi-step ground and excited state absorption and energy transfer processes of Yb^3+^ sensitizer (*S*) and Er^3+^ activator ions resulting in activator upconversion with simultaneous energy deactivation, non-radiative quenching by cross-relaxation (CR) and multiphonon relaxation (MPR) quenching, as well as energy migration and storage in the different excited states of the numerous sensitizer (*S*) and activator ions^14–17^. These activators involved in the abovementioned processes within each single *D* nanoparticle are the actual donors (*D*_*i*_). This complexity is well evidenced because luminescence lifetimes (rise- and decay times) and intensity, as well as upconversion quantum yield, depend on excitation power, and the composition of UCNPs^14,17–19^. In a single UCNP, hundreds up to thousands of *D*_*i*_ species (e.g., Er^3+^ activators) can be found, and are distributed over the entire volume of the nanoparticle, typically 10–25 nm in diameter. Due to the short Förster distance (*R*_0_ ≈ 2–6 nm)^20^, however, only the superficially localized *D*_*i*_ are capable of non-radiative energy transfer to the surface-bound *A* molecules, while the *D*_*i*_ localized beyond R_0_ in the center are not directly sensitive to their presence^17,21–23^. The energy storage at the long living excited states of the *S* network, on the other hand, enables continuous “recharging” of the superficial ground state *D*_*I*_^12,14,19,24^ and results in augmented sensitized *A* emission intensity but also diminishes the FRET-associated shortening of luminescence lifetime of *D*_*i*_ in the presence of surface-bound or *A*^17^. Moreover, due to the inorganic nature of UCNPs, new challenges emerge in rendering them bio-responsive. The surface modifications for functionalization of UCNPs and conjugation of biorecognition molecules inevitably increase the *D – A* distances to concerning R_0_, and in consequence, reduce RET efficiency and the sensitivity of both the decay and intensity based responses towards the surface-bound *A* molecules^25^.

**Fig. 1:**
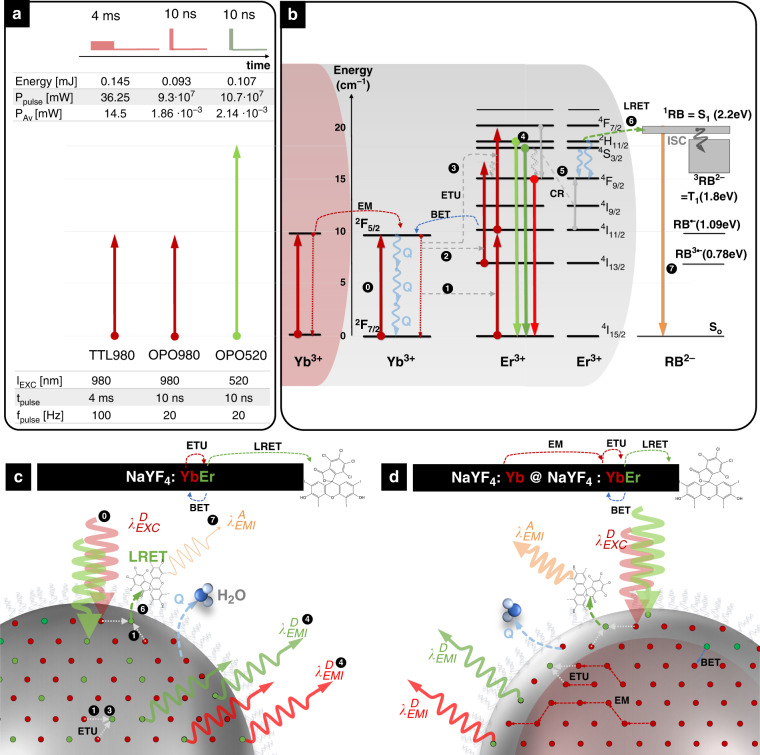
The excitation schemes and mechanism of UC-LRET between Yb/Er donor NPs and Rose Bengal acceptors. **a** Three different excitation schemes were used, i.e., (TTL980) 4 ms pulse from CW 980 nm laser diode controlled with TTL, (OPO980) 10 ns pulse of 980 nm from OPO, and (OPO520) 10 ns pulse of 520 nm from OPO. **b** Energy scheme and transfer in Yb^3+^/Er^3+^—RB system (energy level for RB taken from ref. ^49^); ETU energy transfer upconversion, EM energy migration, BET back energy transfer, ISC inter system crossing, CR cross-relaxation, Q quenching, LRET luminescence resonance energy transfer, schematic presentation of (**c**) YbEr core-only (gray area) UCNP donor nanoparticle compared with (**d**) core (reddish area)-shell (gray area) Yb@YbEr donor NPs, both with RB on the surface. Arrow thickness is qualitatively proportional to the intensity;  – Er^3+^ ions,  – Yb^3+^ ions

Although there are examples where *D* nanoparticle steady-state emission responds to *A* presence^10,23,26^, these evidence do not unambiguously confirm the non-radiative nature of *D* – *A* energy transfer. For this reason, monitoring the *D* luminescence kinetics (expected to be shortened upon non-radiative the RET process to *A)* is considered the most reliable indicator of the non-radiative energy transfer mechanism. However, with the current designs of the UCNPs, the luminescence decay of the *D*_*i*_ depends also on concurrent migration of the excitation energy stored in the long-lifetime excited states of the sensitizer network and consequently on the excitation pump power and pulse length^14^, which can hamper the reliability of the luminescence decay as RET indicator. For this reason, the impact of the photoexcitation scheme and UCNPs chemical architecture on the luminescence kinetics of *D*_*I*_ in response to the presence of *A* molecules on the surface of UCNPs was evaluated (Fig. 1). In particular, three types of laser pulses were compared for two types of UCNPs architectures, both experimentally and by numerical modeling with differential rate equations (DRE). We compared different merit indicators for RET efficiency and observed luminescence intensity kinetics of upconversion luminescence RET (UC-LRET) systems for long (4 ms pulse, 6 ms break, f_REP_ = 100 Hz) and short (10 ns, f_REP_ = 20 Hz) 980 nm photoexcitation pulses targeting the sensitizing Yb^3+^ ions, as well as for short (10 ns, f_REP_ = 20 Hz) lasting 520 nm photoexcitation pulses targeting directly the activator–donor Er^3+^ ions (to avoid Yb–Yb energy diffusion within sensitizer network). These three photoexcitation schemes were compared for two different UCNP nanoarchitectures, core-only YbEr (NaYF_4_: 20% Yb, 2% Er) and core-shell Yb@YbEr (NaYF_4_: 20% Yb @ NaYF_4_: 20% Yb, 5% Er) for rising concentrations of RB acceptor bound on their surface. In addition, the steady-state luminescence spectra with 980 nm continuous wave (CW)—photoexcitation were measured for both donor types. These two compositional architectures were purposefully selected, the first being gold standard upconverting nanoparticles and a reference point, the second one as a new version of the YbEr co-doped nanoparticles, previously optimized for UC-RET sensing^17^. Throughout the manuscript, we use three different terms, i.e., RET, FRET, and UC-LRET, to describe specific characteristics of (1) the general Förster mechanism of resonant energy transfer between excited donor and ground state acceptor (RET), (2) conventional RET between molecular donor and acceptor fluorophores (FRET), and (3) RET between upconversion luminescent lanthanide-doped nanoparticle donors and organic acceptor fluorophores (UC-LRET), respectively.

## Results

**Fig. 2:**
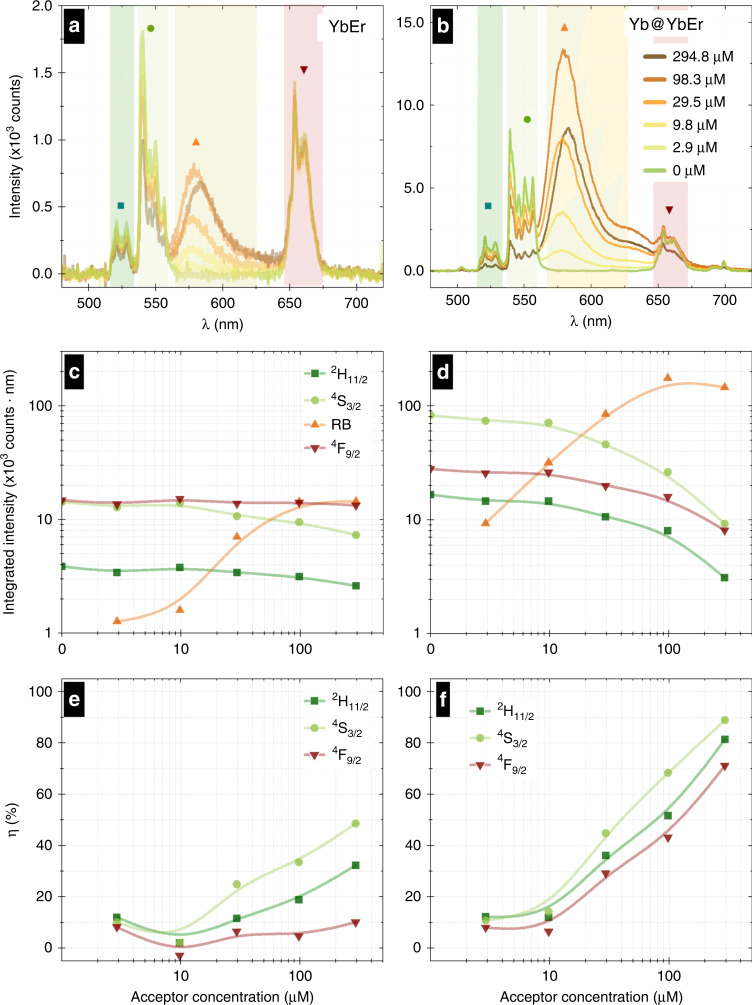
Steady-state spectral properties of YbEr and Yb@YbEr samples undergoing UC-LRET to Rose Bengal acceptor. Steady-state upconversion spectra (980 nm excitation) of **a** core only and **b** core-shell UCNPs with different concentrations of Rose Bengal attached to their surface. Rose Bengal concentration-dependent integrated steady-state emission intensities from ^2^H_11/2_ (), ^4^S_3/2_ (), and ^4^F_9/2_ () levels of Er^3+^ ions as well as sensitized emission intensity from RB () for **c** YbEr and **d** Yb@YbEr, respectively. RB concentration-dependent UC-LRET efficiency for **e** YbEr and **f** Yb@YbEr, respectively, were calculated from intensity changes of different donor emission bands in response to acceptor presence. Integrals are based on spectral ranges indicated in **a**, **b**

We started our studies with the characterization of the two architectures in steady-state excitation conditions (Fig. 2). We took special care to anchor the RB dye directly to the surface of UCNP and compared the result to a control experiment, where the surface was blocked from the binding of the RB dye (Rose Bengal dye titration section in SI), aiming to understand and confirm that non-radiative process is involved upon anchoring the RB dye in clear contrast to radiative reabsorption. By titrating the concentration of RB over a wide range, we were able to observe how the given NP responded to variable RB acceptor concentration, which also enabled us to see which composition resulted in the highest sensitivity for small concentration and also what was the maximal RET sensitized acceptor response available compared to UCNP only signal. Figure 2 shows un-normalized emission spectra of the same mass concentration of YbEr and Yb@YbEr NPs with rising concentrations for RB molecules (Fig. 2a, b, respectively). Based on the steady-state spectra, the changes of emission intensities integrated over wavelength ranges for Er^3+^ energy levels (^2^H_11/2_ + ^4^S_3/2_, ^4^F_9/2_) and Rose Bengal emissions were quantified and presented for YbEr (Fig. 2c) and Yb@YbEr (Fig. 2d). Finally, the UC-LRET efficiency was estimated (Fig. 2e, f, for YbEr and Yb@YbEr nanoparticles, respectively) based on the equation Eq. 1 (a more detailed explanation of RET formalism can be found in SI, Fig. S1 and Eq. S1–S9)

1 $$\eta = 1 - \frac{{I_{DA}}}{{I_D}}$$

where *I*_*D*_ and *I*_*DA*_ denote the integrated luminescence intensity of the donor NP only and the donor NP covered with acceptor molecules, respectively, calculated separately for the spectral range of 516–533 nm (Er^3+^ emission from ^*2*^*H*_*11/2*_), 537–560 nm (^*4*^*S*_*3/2*_) or 646–674 nm (^*4*^*F*_*9/2*_).

**Fig. 3:**
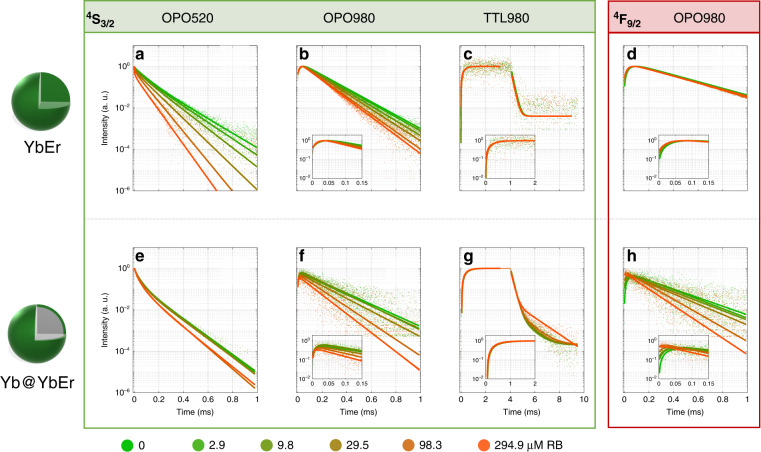
Qualitative comparison of luminescence kinetics of YbEr and Yb@YbEr samples undergoing in UC-LRET. Luminescencekinetics for emission of energy levels. ^4^S_3/2_ (525–550 nm) and ^4^F_9/2_ (650 nm) emission from (**a**–**d**) YbEr core only and (**e**–**h**) Yb@YbEr core-shell UCNPs, respectively, at a rising concentration (0, 2.9, 9.8, 29.5, 98.3, and 294.8 µM) of Rose Bengal attached on their surface. Various excitation modes were compared: **a**, **e** 520 nm 10 ns pulse from OPO laser; **b**, **d**, **f**, **h** 980 nm 10 ns pulse from OPO laser; **c**, **g** 980 nm 4 ms pulse from TTL controlled 10 W CW laser diode. Additionally, luminescence decays of ^4^F_9/2_ level emission of Er^3+^ under 980 nm with 10 ns pulse from OPO are shown (**d**, **h**). The insets contain magnified rise time regions on the corresponding kinetic profiles. A complete set of data for green and red emissions is presented in SI

To understand the role of donor nanoparticle architecture and the excitation path of the donor, the luminescence kinetics of the UC-LRET system was studied for the two types of donor nanoparticles and rising RB concentrations (Fig. 3). Because direct excitation (short wavelength in the range 350–550 nm) of the *D* ions brings the risk of high background signal (e.g., autofluorescence and scattering of light in the sample), the energy-transfer upconversion obtained through indirect photoexcitation at NIR spectral region is strongly preferred for practical bioassay applications. To make the comparison versatile, we have also studied the luminescence kinetics using a short pulse of OPO laser in Stokes and anti-Stokes modes. Direct Er^3+^ photoexcitation at 520 nm was achieved in Stokes mode with 5–7 ns OPO pulses (20 Hz repetition, median energy 0.107 mJ). The anti-Stokes mode exploited indirect photoexcitation of Er^3+^ ions through 980 nm photoexcitation of sensitizer Yb^3+^ ions under high energy 5–7 ns OPO pulses with 20 Hz repetition (median energy 0.093 mJ). In addition, 4 ms long TTL pulses (10 ms break between pulses) were used to trigger CW laser diode (median value of energy 0.145 mJ) to record the transients of UC luminescence until saturation, i.e., the kinetic balance of the UC emission intensity.

While a combination of the ground and excited state absorption (GSA + ESA, respectively) in Er^3+^ under 980 nm excitation is possible, it was estimated to be ca. 100-fold weaker as compared to Yb^3+^ sensitized energy transfer upconversion (ETU) (2 Yb* + Er → 2 Yb + Er*, where ‘*’ indicate ion in an excited state)^7,27,28^ because of the higher absorption cross-section of Yb^3+^ at 980 nm compared to Er^3+^ and the longer luminescence lifetime of ^2^F_5/2_ level of Yb^3+^ (>500 μs) compared to ^4^I_11/2_ of Er^3+^ (ca. 250 μs). Further, the rise times of Er^3+ 4^S_3/2_ and ^4^F_9/2_ emissions under 10 ns short-pulse excitation at 980 nm (Fig. 3b, d, f, h) indicate the involvement of ETU. The role of Yb^3+^ ions is thus critical to achieving efficient upconversion. Still, it simultaneously brings issues related to energy migration and the ‘recharging’ effects, which hinder the use of luminescence kinetics for the quantification of UC-LRET.

**Fig. 4:**
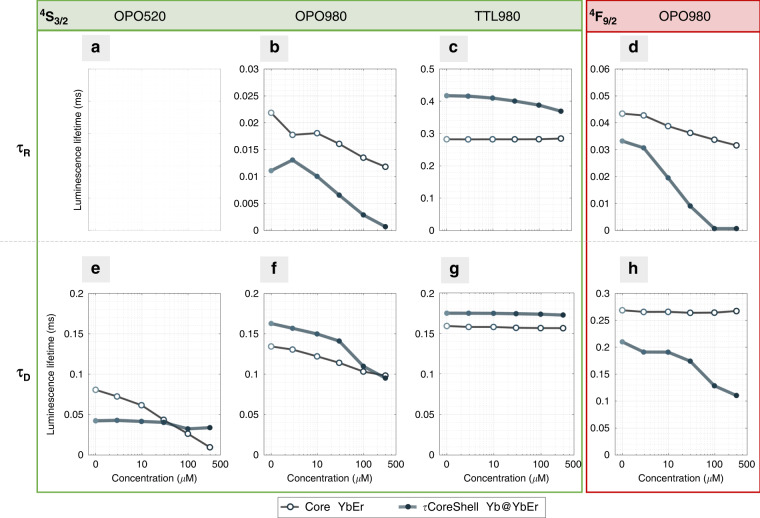
Quantitative comparison of luminescence rise and decay times of YbEr and Yb@YbEr samples undergoing UC-LRET. Luminescence rise and decay times for energy levels. ^4^S_3/2_ (525–550 nm) (**a**–**c**, **e**–**g**) and ^4^F_9/2_ (650 nm) (**d**, **h**) emission from (open symbol) YbEr core only and (solid symbol) Yb@YbEr core-shell UCNPs respectively, at a rising concentration of Rose Bengal attached to their surface. Various excitation modes were compared: **a**, **e** 520 nm 10 ns pulse from OPO laser; **b**, **f** 980 nm 10 ns pulse from OPO laser; **c**, **g** 980 nm 4 ms pulse from TTL-triggered 10 W CW laser diode. The luminescence rise times (τ_R_) (**a**–**d**) and averaged decay (τ_D_) (**e**–**h**) components are shown separately

Figure 3 shows experimental luminescence kinetic profiles *I(t)* vs. RB concentration, and these data were further analyzed, namely the acceptor concentration-dependent luminescence rise (τ_R_) and decay (τ_D_ stands for single or averaged decay component) time values (Fig. 4) were extracted from these kinetic profiles with methods described in SI section V (Figs. S6, S8, S9, S11). Additionally, UC-LRET efficiencies *η* (Eq. 2) based on the rise and decay times for ^4^S_3/2_ (Fig. S8) and ^4^F_9/2_ (Fig. S11) emissions are shown in Figs. S7, S10, respectively.

2 $$\eta = 1 - \frac{{\tau _{DA}}}{{\tau _D}}$$

Where *τ*_*D*_ and *τ*_*DA*_ denote luminescence rise time (when rise times are considered) or the luminescence lifetime (when decays are considered) of the donor NP only, and donor NP covered with acceptor molecules, respectively. Additional information about the analysis of luminescence kinetic profiles (separately for the rise and decay lifetimes) is described in equations S7, S8, and S9.

In contrast to the OPO520 photoexcitation, in the OPO980 scheme, the Er^3+^ donor ions are excited indirectly by pumping the sensitizer Yb^3+^ ions, whose energy is transferred to the Er^3+^ activator in a set of energy cross-relaxations, energy migrations, and energy-transfer upconversion processes. Because the interaction between sensitizers and activators, both in terms of excitation and luminescence emission, occur simultaneously and the rise times are relatively fast, it is impossible to reliably fit these data individually in the rising and decaying part of the kinetic curve. Therefore, we have implemented a simplified three-level upconversion DRE model, which enabled us to simultaneously fit the rising and decaying parts of the kinetic profiles (Table S2). Although time-consuming, such a DRE fitting approach generated more reliable and coherent emission rates than fitting rise- and decay- parts individually. This approach was applied for both the ^4^S_3/2_ and the ^4^F_9/2_ emission profiles (Tables S3 and S4) and used to calculate the efficiencies (Tables S5,S6). The luminescence acquired for a long 4 ms photoexcitation pulse in the TTL980 excitation scheme displayed separate exponential growth and decay parts. Accordingly, they were fitted independently for the rise and decay parts of the kinetic profile with analytical exponential expressions. Moreover, where it was necessary, the decaying luminescence exploited the bi-exponential model, where the longer component is assumed to originate from the “radiative” interior of the donor nanoparticles from ions, which are not exposed to surface acceptors or quenchers. In contrast, the short component can be related to surface Er^3+^ ions, which are more exposed to quenching or RETing to acceptors.

### Results based on the theoretical model

To further understand the behavior of the system, we adopted the DRE model of upconversion in the YbEr system. The model purposefully considered only LRET from Er^3+^ to RB singlet state but not from Er^3+^ to RB triplet state. The model included a complicated “machinery” responsible for efficient upconversion in the YbEr system. The performed simulations were capable of generating upconversion luminescence kinetics of Yb^3+^ sensitized energy transfer upconversion of Er^3+^ ions. With these simulations, we managed to qualitatively distinguish between different excitation schemes, i.e., between (i) 10 ns pulse triggered 520 nm OPO laser pulse, (ii) ca. 10 ns pulse triggered 980 nm OPO laser pulse, and (iii) TTL triggered 4 ms 980 nm CW laser diode pulse. All these simulations were performed with the same rates in DRE equations. At the same time, only excitation pulse intensities and pulse lengths differed from reflecting the specific features of the OPO520, OPO980, and TTL980 excitation schemes. Moreover, the luminescence rise- and decay times in luminescence kinetics responded proportionally to variation in acceptor concentration.

**Fig. 5:**
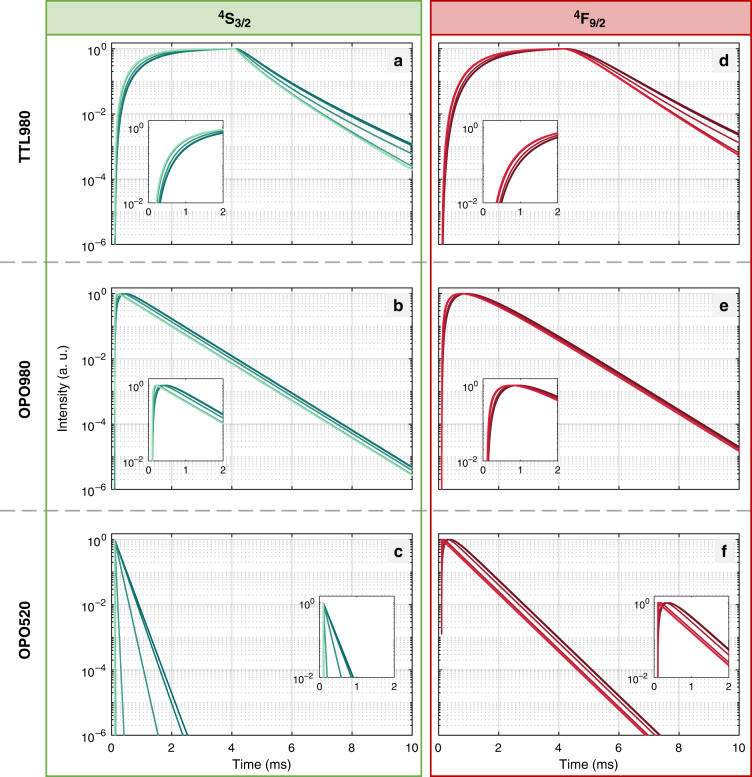
**Luminescence kinetics for Yb-Er donor and RB acceptor UC-LRET system based on a theoretical model of differential rate equations.** The acceptor concentration-dependent kinetics of ^4^S_3/2_ level (left column: **a**–**c**) and ^4^F_9/2_ (right column **d**–**f**) of Er^3+^ derived with DRE modeling for the three excitation schemes: TTL triggered 4 ms 980 nm CW laser diode pulse (top row, **a**, **d**), ca. 10 ns 980 nm OPO laser pulse (middle row, **b**, **e**) and ca. 10 ns 520 nm OPO laser pulse (bottom row, **c**, **f**). The acceptor concentration increases from the darkest (no acceptor present) to the lightest color (highest concentrations; details of the model in SI)

Complex interdependent ET mechanisms among sensitizer and activator ions within the UCNPs cannot be easily modeled because excitation regime, relaxation rates, and energy transfer between long-living manifolds are related to (in a nonlinear manner) power density, laser power, and excitation duration as well as with absolute and relative concentration of lanthanides sensitizers and activators^14^. Our simulations were based on phenomenological parameters of respective rates and supporting descriptions of provided calculations (see SI—Tables S7, S8, Figs. S13, S14, and Eq. S10–S16), and they produced kinetic luminescence profiles comprising rise and decay luminescence components, which were analogous (Fig. 5) to the kinetic profiles observed experimentally (Fig. 3). These results confirmed, that although rate equation modeling is an averaged approach, the developed model was indeed phenomenologically properly describing of our system behavior and let us discuss the relationships found in the course of the studies.

## Discussion

The in situ RET-based bioassays are an important tool in biomedical research and diagnostics. Still, they suffer from some technical and spectroscopic challenges with the conventionally used molecular fluorophores. The efficient, narrowband, and long-living anti-Stokes emission found in lanthanide-doped upconverting nanoparticles can reduce the background signal and enhance sensitivity. Therefore, based on our previous work, the major motivation of current studies was to understand how the photoexcitation mode of Er^3+^ donor ions (i.e., continuous/long-pulse vs short-pulse photoexcitation and direct vs. upconversion mediated excitation) affect the responsiveness of Er^3+^ emission in green (^4^S_3/2_) and red (^4^F_9/2_) channels to the presence of acceptor molecules (Rose Bengal) anchored on the UCNPs of two different compositional architectures (core only 20Yb2Er vs. core-shell 20Yb@20Yb5Er). The 20Yb2Er sample was selected to be as a reference material—the most well-known upconverting material that could be a first choice material for UC-RET detection, while the 20Yb@20Yb5Er presents a new, optimized compositional architecture scheme^17^. To compare the two samples with different compositions, the relative impact of RB on the luminescence intensity and kinetics of *D* – *A* versus *D* within each sample was used as figure merit.

### Steady-state spectral response to acceptor

Studying steady-state spectra (Fig. 2), higher upconversion intensity and more efficient energy transfer from Er^3+^ donors to acceptor molecules were found for Yb@YbEr architecture compared to YbEr^17^. From the virtual nanoparticle model (VNP) developed in reference^17^, one may estimate that the single NaYF_4_: 20% Yb, 2% Er nanoparticle of 24 nm diameter contains ~45,500 Yb^3+^ and 4550 Er^3+^ ions, while the single NaYF_4_: 20% Yb @ NaYF_4_: 20% Yb, 5% Er core-shell nanoparticle (core diameter equal 24 nm and shell thickness equal 2 nm) contains around 45,500 Yb^3+^ ions in the core, ca. 43,300 Yb^3+^ and ca. 10,800 Er^3+^ ions in the shell, which are 2 and 2.5 more for Yb@YbEr vs. YbEr, respectively for Yb^3+^ and Er^3+^ ions. Concurrently, not only emission intensity from the Yb@YbEr was ca. 7-fold brighter than YbEr, but also the ratio of sensitized RB acceptor to Er^3+^ donor intensity was stronger in the Yb@YbEr sample, although the RB was titrated over the relevant range of concentrations to achieve the optimal RB density on each NP. At the same time, a relatively more significant proportion of Er^3+^ ions are exposed to the acceptor anchored to the surface of the Yb@YbEr as compared to the YbEr NPs. Namely, the Er^3+^ ions in the center of NP of the YbEr do not contribute to sensitized acceptor excitation through LRET. However, their emission may still be absorbed in high RB concentrations and result in radiative ET to the acceptor. Therefore, a control experiment was performed using the same range of RB concentrations, but blocking the NP surface from RB binding. The results confirmed the strong distance dependency of the sensitized acceptor emission as no significant sensitized emission was observed with the studied RB concentration range when the NP surface was blocked.

The preliminary conclusion that, at least in the Yb@YbEr sample, non-radiative processes must be involved was justified by the different quenching of the UCNP emission at 540 nm in YbEr and Yb@YbEr (Fig. 2b). In Yb@YbEr, the Er^3+^ content is higher (5%), and also all the Er ^3+^ are in the shell, i.e., closer to the surface, which causes more efficient Er-Er energy migration and susceptibility to surface quenching, which seems like a steep initial decrease in the donor decay. However, part of the Er^3+^ excited state energy is also transferred by back energy transfer (BET) from ^4^I_11/2_ of Er^3+^ to ^2^F_5/2_ of Yb^3+^ in the core, resulting in an energy migration path that is apparently elongating the lifetime component in the Er^3+^ decay and thus, effectively, making it less susceptible to RET. In YbEr, the Er content is lower (2%), resulting in the Er-Er energy migration being less efficient, and the surface quenching does not shorten as much decay of the donor only. In case only radiative ET (i.e., reabsorption of Er^3+^ emission by RB) is present, the quenching should be close to the same. Moreover, in Fig. 2b, the integrated emission of sensitized acceptor emission is higher than the integrated donor emission without acceptor. This should be impossible if the detector response yield is about the same at 550 and 600 nm. Hypothetically, this could be another proof of rapid cycling and “recharging” of Er^3+^ ions relaxed upon RET, i.e., efficient RET from Er^3+^ to RB and subsequent immediate RB emission enables a new, more rapid radiative relaxation path (indirectly through RET to RB) than present in UCNPs without acceptor (where surface non-radiative quenching may occur). In consequence, we hypothesize RET from Er^3+^ to RB could increase the proportion of excited state energy relaxed via radiative paths compared to non-radiative paths. In such a situation, the total upconversion quantum yield (UC-QY) of the system could also be expected to increase, i.e., more upconverted emitted photons could be generated from the same number of absorbed photons by adding an acceptor coupled to UCNPs by efficient RET compared to a situation without acceptor. Qualitatively similar conclusions about enhanced emission induced by FRET were drawn by Lakowicz et al.^29^ in FRET system with low quantum yield, long-lifetime donor, and short-lifetime acceptor. This FRET system is equivalent in those characteristics to UCNP donor and RB acceptor. Unfortunately, these UC-QY measurements are technically challenging because UC-QY is pump power dependent, lanthanides exhibit low absorption, and UC-QY is typically very weak (<1%), thus suffering from poor accuracy to reliably support the proposed explanation. Additionally, the RB emission is composed of two emission bands. There are also bands at 570 and 620 nm in the absorption spectra, which likely originate from monomer (shorter wavelength) and dimer (longer wavelength) forms of the RB dye, respectively^30^. Monomeric and dimeric fractions are not influencing quantum yields for RB^31^. Further, either homo-RET (i.e. intramolecular RET between RBs densely bound on the surface) or inner-filter effect (i.e., absorption of the light emitted by the RB molecules present at high concentration due to their absorption) is observed for the highest RB concentration resulting in a decrease the RB intensity and apparent redshift of its emission (Fig. 2a, b).

The decrease of the green emission from the ^2^H_11/2_ and ^4^S_3/2_ energy levels of Er^3+^ ions correlates well with the rising concentration of RB acceptor changes (Fig. 2). Interestingly, the Yb@YbEr also shows a significant red emission quenching with rising RB concentration. In opposite to the Yb@YbEr sample, the intensity of red emission of Er^3+^ ions (^4^F_9/2_ → ^4^I_15/2_) of the YbEr sample is stronger and independent of acceptor concentration (Fig. 2c). These differences between YbEr and Yb@YbEr in the proportion and the quenching of the red and green emission originate most probably from the differences in excess of Yb^3+^ sensitizer and density of superficial Er^3+^ activator, which affect the rates of ETU and energy migration mechanisms, and are inherent properties of the given UCNP architectures^19,32,33^. However, the question of why the red 650 nm emission properties in Yb@YbEr nanoparticles are susceptible to RB concentration (Figs. 2b, d, 3h) is especially intriguing because the ^4^F_9/2_ energy level does not fall into the absorption band of singlet state of RB molecule. Notably, the same is not observed for the red emission band in YbEr, which can be most probably explained by the fact that the green emitting of ^4^S_3/2_ + ^2^H_11/2_ level of Er^3+^ is an intermediate step in both 2- and 3-photon excitation pathways of the red-emitting ^4^F_9/2_ level. In the YbEr sample, a significant fraction of Er^3+^ donor ions are in the particle core, where their ^4^S_3/2_ + ^2^H_11/2_ levels are not affected directly by RET thus, the red ^4^F_9/2_ emission intensity is not influenced by RB, and no signs of RET is observed (Fig. 2c, e). However, in the Yb@YbEr sample, the situation is different, as the ^4^S_3/2_ + ^2^H_11/2_ levels of Er^3+^ ions in the shell participate in RET, and the excitation of the red-emitting ^4^F_9/2_ level is therefore affected (Fig. 3d) at an increased concentration of acceptor. It is also important to note that YbEr has 2% Er^3+^ while the shell of Yb@YbEr contains 5% of Er^3+^. The higher concentration of Er^3+^ ions in the Yb@YbEr sample must mean that the Er → Yb and Er → Er energy transfers are more effective and rapid. With 5%, the Er → Er energy transfers likely strongly shorten the intrinsic lifetime of the red-emitting ^4^F_9/2_ level and render the red emission to follow the intensity and the decay of the energy-feeding green emitting ^4^S_3/2_ + ^2^H_11/2_ levels (Fig. 3h).

The other possible explanation for the quenching of the red emission could be the resonance of the red-emitting state to the triplet state of the organic acceptor^34^ Typically, singlet energy level dominates in the FRET process, but in multi-chromophoric systems, singlet-triplet annihilation can play a significant role, especially for single-molecule spectroscopy and lower dependency from power density changes^35^. This, however, should be observed experimentally as quenching of the red emission in both YbEr and Yb@YbEr, which was not the case. Thus, for modeling, we considered the interaction of the ^4^S_3/2_ + ^2^H_11/2_ level with the singlet state of the RB molecules only.

### Luminescence kinetics response to acceptor

Despite the fact that significant quenching of the donor and increase in the sensitized acceptor emission intensities were observed for rising concentration of acceptor, the steady-state spectra as such, are not capable to distinguish between the non-radiative and radiative energy transfer mechanism and studying the luminescence kinetics is considered to be the only unambiguous evidence for non-radiative RET with a strong dependency on the donor-acceptor distance. While qualitative comparison of luminescence kinetics (Fig. 3) gives a good overview of how the system responds to acceptor, quantitative evaluation of luminescence rise and short/long decay components (Fig. 4) and thereof derived UC-LRET efficiencies (Figs S7,S10) allow to better conclude about the suitability of particular materials and excitation scheme for practical implementation.

In luminescence kinetics mode, the direct short-pulse photoexcitation at 520 nm (OPO520) surprisingly results in a more significant decay response with the conventional YbEr sample to the RB acceptor concentration, but in contrast, with indirect short-pulse 980 nm excitation (OPO980) the Yb@YbEr performs better (Fig. 3). Basically, under 520 nm excitation, the Yb@YbEr should behave at least equally well to the YbEr, unless the Er → Yb back energy transfer (BET), the Yb → Yb energy migration and energy storage in the Yb^3+^ network, and Er → Er energy-transfer and cross-relaxations are more dominant in the core Yb@YbEr due to excess of Yb^3+^ and higher (compared to YbEr sample) Er^3+^ concentration in the shell. Thus, the faster decays of donor emission under OPO520 in Yb@YbEr without the acceptor are likely due to 5% Er instead of 2% Er and indicate the importance of Er γ Yb BET or Er-Er CR/EM. This can also be the reason for a less significant decay response with Yb@YbEr upon RB^16^. Additionally, the results presented in Fig. 3e vs. Fig. 3a is a direct proof that the BET is present and efficient, and the energy storage in the Yb^3+^-network (without Er^3+^) results in a slower decay response even if the Er^3+^ are closer to the acceptor. The effect of energy storage, migration, and “recharging” has also been noticed previously^17^, and evidenced with current experiments in a more clear way as diminishing the change of the donor decay in Yb@YbEr upon long-pulse 980 nm excitation (TTL980) (e.g., Fig. 3f, h vs Fig. 3g, and corresponding panels in Fig. 4 and in SI—Fig. S6,S9). Therefore, in Yb-Er (or similar) co-doped systems, Yb^3+^ ions are not only responsible for (i) efficient absorption of NIR radiation and (ii) sensitization of upconverted emission ETU but are also involved in energy redistribution by (iii) filtering excited state energy from Er^3+^ ions by Er → Yb BET, (iv) energy storage at long-lifetime excited state of Yb^3+^, (v) energy balancing by Yb → Yb → … → Yb → Yb EM, and (vi)“recharging” of the Er^3+^ by Yb → Yb → … → Er ETU. In consequence, the spatial distribution of both Yb^3+^ and Er^3+^ and concentration of Er^3+^ all have an important role in the responsivity of Yb^3+^, Er^3+^ based UCNPs to the acceptors on their surface (Fig. 1c, d).

The experimental data with OPO520 excitation (Figs. 3, 4 supported by Figs. S6–S11), were fitted with a double exponent tail fitting procedure (Table S2), and the two exponents were ascribed to two classes of Er^3+^ ions – those superficial, which are susceptible to the presence of acceptor, and those in the center volume, which are protected from surface quenching and should be inert to the presence of the surface-bound acceptor due to *D* – *A* distances beyond R_0_. Interestingly, based on OPO520 results from Fig. 4e, we suppose that independently from the *D* – *A* distance, the excited state energy of the Er^3+^ ions in the entire YbEr nanoparticle volume could effectively be channeled through Er-Er EM to the superficially located Er^3+^ ions which stay within Förster distance to the RB acceptor and thus the whole NP could indirectly ‘contribute’ to the RET. The results indicate that the averaged decay of the classic YbEr core UCNP was responsive to acceptor presence under 520 nm photoexcitation. Because respective energy levels of Yb^3+^ and Er^3+^ ions match each other well, e.g. the energy gaps Er^3+^: ^4^I_11/2_ – ^4^I_15/2_, ^4^I_15/2_ – ^4^F_9/2_, ^4^I_11/2_ – ^4^F_7/2_ and Yb^3+^: ^2^F_5/2_ – ^2^F_7/2_ are similar, the absorbed energy may be transferred between these levels and these ions back and forth. Therefore, even under OPO520 (capable to photo stimulating only Er^3+^ ions), the mentioned processes are known to lead to Er → Er energy migration (EM), Er-Er cross-relaxations (CR, Fig. 1b), as well as Er → Yb energy transfer (BET, Fig. 1b). Because there are 4-times more Yb ions than Er in the shell of the Yb@YbEr sample, the latter process should be followed by energy “trapping” by EM between long-living Yb ions in the Er^3+^ free core for a significantly long time as the Yb relaxation by ETU to Er is missing. Thus, the Yb excited state lifetimes in the 20% Yb-only core must be significantly longer than in the 20%Yb2%Er shell, even the Yb–Yb EM rate is the same. We expect that the back energy transfer and the energy storage in the Yb^3+^ network are resulting in delayed ‘recharging’ of the superficial Er^3+^ ions and stand behind the weak decay response of the green Er^3+^ emission observed with Yb@YbEr in the presence of acceptor using OPO520 excitation. We also hypothesize the whole UCNP volume may effectively interact with surface quenchers through complex, collective, and history-dependent energy transfer processes found in co-doped systems^14,36,37^. We suppose there can be fast energy diffusion between the ions in the same nanocrystal, which resembles the observation of molecular lanthanide networks and a phenomenon called co-luminescence^38^.

Using indirect excitation of Er^3+^ donors via Yb^3+^ sensitizers with OPO980 or TTL980 also luminescence rise times are observed, which are primarily a consequence of the energy-transfer upconversion process, which are additionally affected by the concurrent interdependent excited state absorption, energy-transfer, and both radiative and non-radiative relaxation processes^14^. Despite the same excitation wavelength, the TTL980 and OPO980 excitation schemes differ in the resulting temporal population of Yb and Er excited states and subsequently on relative contributions of different energy transfer and relaxation processes that, in consequence, affect the course of the sensitized Er^3+^ emission being quenched by acceptor molecules. The rise times are dependent on the lifetimes of the intermediate and/or upper levels and sequential, step-by-step process in populating the emitting level. Therefore, the slow rise times may provide additional information on how the building-up of the Er^3+^ emitting state population is affected by the additional non-radiative relaxation pathway introduced by RET. Moreover, when the rise times are comparable to the decays, it becomes apparent these two processes are intertwined and thus difficult to split and analyze with conventional methods. It is also quite understandable that these long time-scale processes shall depend on the excitation pulse intensity and excitation timescale—depending on the kinetic balance of the system reached during the excitation pulse. The RB concentration-dependent rise times that have indeed been observed here are, most probably an additional (however not previously explored in scientific literature) merit indicator for the UC-LRET process. The luminescence rise time variation upon RB presence is especially significant for both (green and red) Er^3+^ emission of core-shell Yb@YbEr under indirect short pulse 980 nm photoexcitation (OPO980). The luminescence rise time in UC-LRET has not been considered so far as a reliable figure of merit, but our results indicate that with OPO980 excitation, the τ_R_ parameter at the red emission channel (Fig. 3 and S11) demonstrates the highest sensitivity to the RB acceptor concentration both with YbEr and Yb@YbEr samples - being at comparable to the sensitivity of averaged lifetime τ_D_ for YbEr with OPO520 excitation. The rise times of YbEr and Yb@YbEr samples under OPO980 excitation respond to the RB acceptor concentration both at the green and red emission channels (Figs. 3,4) even in YbEr samples, where the decay of red Er^3+^ emission does not change at all with increasing RB concentration.

In general, the luminescence kinetics of the Yb@YbEr sample is more responsive to the RB acceptor concentration with indirect, short pulse OPO980 excitation of the Er^3+^ as shown in Figs. 3,4, and to a minor extent also with TTL980 excitation. In contrast, the YbEr sample is clearly more responsive to direct, short-pulse OPO520 excitation. The luminescence rise- and (single / averaged) decay components were subsequently used to derive (using Eq. 2) efficiencies of RET between Er^3+^ ions and RB acceptor molecules (Figs. S7,S10). The complete set of data (decay curves, decay values, and the herein derived UC-LRET effectiveness) for the green and red decays in various excitation configurations are further provided in SI section V. All these results once again show the YbEr sample outperforms the Yb@YbEr sample only in the OPO520 excitation regime by 2-fold, but it must be noted that it is 10-fold dimmer than the Yb@YbEr.

Considering the biomedical applications, the OPO520 and OPO980 excitation are technically demanding to be realized in point-of-care (POC) diagnostics devices. Further, with OPO520, it is evident that the advantages gained by anti-Stokes emission are not available. The use of laser diodes (LD) excitation sources (instead of the solid-state OPO laser or similar) offers a much cheaper, more robust, and miniature solution. Such laser diodes provide sufficient excitation power to induce upconversion emission in CW or TTL long (ca. a few ms) pulsed modes. In the TTL980 photoexcitation scheme, with long (4 ms) pulse photoexcitation, the photoexcitation energy can be accumulated in the long-living excited states of Yb^3+^ ions and in the Yb^3+^ network, which, even after the pumping is finished, is capable to migrate and progressively repopulate the Er^3+^ activators, i.e. the actual donor ions, rapidly after they have transferred their excited state energy to acceptor molecules by LRET and returned to their ground state^14,17^. The energy migration in such a network can propagate over long distances, e.g., 140 nm in Gd^3+^ or up to 400 nm in 40% Yb^3+^ migration network was estimated, which is 7 and 20 fold the diameter of typical UCNP^20,39^. It is important to add that the significance of temporal energy storage in the sensitizer network and subsequent likelihood for “recharging” of donor ions will most probably increase with laser pump power. Moreover, the consequences of such recharging shall make the luminescence kinetics worthless for the analysis of UC-LRET efficiency. Concerning the upconversion LRET, we should state that although indirect 980 nm excitation is effective in steady-state, the TTL980 excitation was disappointing as neither rise nor decay times were sufficiently susceptible to the concentration of the RB acceptor molecules.

### Analysis of the differential rate equations

The adopted DRE model enables to predict and further simulation in silico (Fig. 5) of how the luminescence rise and decay times respond to various excitation schemes even the processes are intertwined. Because upconversion is a complex system, such modeling has not only helped us to solve the hesitation about the nature of red Er^3+^ emission susceptibility to the acceptor but also shows promise to further optimize the architectural composition of UCNPs to improve their responsiveness in UC-LRET and capability in biosensing applications. In reality, however, the situation is more complex as the ion-ion ET/CR rates in the UCNPs are concentration-dependent (as the distances between ions are different). The increased concentration of Er^3+^erbium ions (5 vs 2%) may increase the ET/CR rates and thus shorten the red emissive state lifetime so much that it follows the green state (the one which actually feeds the red state).

### Summary discussion

In summary, we found interesting relationships, which confirm that specially designed core-shell architectures of UCNPs (where increased concentration donor ions are located only in the shell) are beneficial for UC-LRET sensitivity over conventional UCNPs (where donor ions are evenly distributed in the whole volume). This conclusion is apparently expected, but it does not explain the complicated nature of the discussed phenomena nor support knowledge-based optimization of the compositional architecture of UCNPs as luminescent donors in UC-LRET. Moreover, we demonstrated that the Yb^3+^ ions, although significant for efficient upconversion and intensity of the UC-LRET sensitized acceptor emission, may have their parasitic role in decay-based UC-LRET sensing because they can accumulate and migrate excited state energy, facilitate back energy transfer from the excited Er^3+^ ions and ‘recharge’ Er^3+^ donor ions as soon as they have transferred their energy to acceptor molecules and returned to their ground states. In consequence, the high excess of Yb^3+^ ions may impair the decay response of the UC-LRET system.

The most sensitive UC-LRET quantification based on luminescence kinetics was achieved with the Yb@YbEr sample using OPO980 excitation and the rise times in green (520–540 nm) and red (650 nm) emission channels. However, the wide-pulse TTL980 excitation using TTL-controlled pulses from CW laser diodes would be most desirable from a technology perspective: it is the simplest, most cost-effective, and robust technical solution, but unfortunately, the kinetic UC-LRET response with TTL980 excitation is only barely sensitive to the presence of the acceptor. These studies provided important suggestions, that short powerful excitation pulses at 980 nm and rise times measurements can provide highly sensitive assays and emphasize further the importance of the compositional architecture of the UCNPs as donors to improve either the steady-state luminescence intensity or kinetic responses of UC-LRET-based assays. Actually, it shall be technically easier to measure the rise kinetics compared to the decays in terms of photon budget and absolute signal strength.

The energy storage and migration in the Yb^3+^ sensitizer network, which is facilitated by long-pulse excitation of the Yb^3+^ sensitizers in both YbEr and Yb@YbEr samples, is disadvantageous for the kinetic response of UC-LRET. The possible remedies for these could potentially be (1) use more powerful short-pulse excitation; (2) optimize Yb^3+^ content and Yb^3+^ to Er^3+^ ratio to keep absorption cross-section high but simultaneously limit the energy migration paths; (3) further evaluate the potential of rise kinetics with different excitation intensities and pulse lengths as a new factor to monitor the UC-LRET response; (4) exploit steady-state spectral information with appropriate control experiment for the possible radiative energy transfer; (5) search for spectral/kinetic features of luminescence emission of other energy levels of donor ions (here ^4^F_9/2_ of Er^3+^ ions) as alternative indicators of UC-RET. However, together with the possible Er → Yb back energy transfer and Er-Er cross-relaxation processes, that become augmented at increased concentrations of dopants, the efficient energy balancing in the single nanocrystal may potentially result in that also the excited state energy of the sensitizer and activator ions located beyond the Förster radius in the core of the UCNPs can be relaxed via LRET through the superficial activators. Moreover, a combination of the abovementioned methods could be amalgamated together with sophisticated artificial intelligence-based analysis to draw quantitative conclusions from a set of features rather than just one parameter. Our studies shine new light on the UC-LRET and will help to further optimize the compositional architectures of UC-LRET reporter donor nanoparticles for enhanced UC-LRET sensing.

## Materials and methods

### Chemical synthesis and sample

*Preparation of Core and Core@Shell Material*: In a typical synthesis, the given amounts (2 × 10^−3^ mol Ln^3+^) of (CH_3_COO)_3_Ln precursors were added to the three-neck flask with OA (12 cm^3^) and ODE (30 cm^3^). The solution was stirred under a nitrogen atmosphere and heated slowly to 140 °C, followed by degassing under a vacuum for 30 min to remove oxygen and water. Next, solutions of ammonium fluoride and sodium hydroxide dissolved in methanol were added. After that, the reaction temperature was increased quickly to 300 °C and kept at this temperature for 60 min under a nitrogen atmosphere. After the UCNPs formation, the mixture was allowed to cool to room temperature. The UCNPs were precipitated by adding ethanol and isolated by centrifugation at 10,000 pm for 10 min. For purification, the resulting pellet was dispersed in a minimal amount of *n*-hexane and again precipitated with excess ethanol. The final product was isolated by centrifugation at 14,000 rpm for 10 min and dispersed in 12 cm^3^ *n*-hexane. The final product stabilized with OA ligands was dispersed in 5 cm^3^ of chloroform (CHCl_3_). More details can be found in SI (in section III Materials and methods section with sample characterization (XRD, TEM, EDS) in Fig. S2 and Rose Bengal dye titration in Fig. S3).

### Sample characterization

Spectroscopic measurements were provided with an experimental setup shown in Fig. S4. A detailed description of lasers parameters is shown in Table S1; filters used for lifetime measurements compared with emission of nanocrystals and parameters of RB dyes are shown in Fig. S5. Luminescent properties were measured for colloidal samples in custom-made quartz tubes (1.5 mm light path from the center, with 90^o^ collection configuration) using a home-built setup, more details could be found in SI. The samples were tenfold diluted before the measurements. Spectra of samples were obtained with HR4000 Ocean Optics Spectrometer, and luminescence lifetimes (rise and decay lifetimes) were obtained with a photomultiplier (PMT1001/m, Thorlabs). We used two types of lasers - MDL-F-980-10W (for long-pulse excitation with 980 nm wavelength, controlled with TTL) and tunable laser OPOLLETE 355 LD Optical Parametric Oscillator (OPO) (for short pulse photoexcitation with 520 and 980 nm wavelengths).

### Average lifetimes

When the luminescence was decaying with multiple exponential characteristics, we adopted average luminescence lifetimes, which was calculated with an equation ${\uptau} = \frac{{\mathop {\sum }\nolimits_{{{\mathrm{i}}}} {{{\mathrm{A}}}}_{{{\mathrm{i}}}} \cdot {\uptau}_{{{\mathrm{i}}}}}}{{\mathop {\sum }\nolimits_{{{\mathrm{i}}}} {{{\mathrm{A}}}}_{{{\mathrm{i}}}}}}$, where A_i_ and τ_i_ indicate the amplitude and decay of each lifetime component (i = 1, 2, or higher)^40^. The methods used to analyze the luminescence decays are presented in SI section V, with a description of the differential rate equation model used for the photoexcitation model (Table S2).

### Theoretical modeling

Choice of *D*-*A* pair based on spectral overlap were presented in Fig. S1, and based on previously conducted calculations of *R*_*0*_^17^. To qualitatively understand the impact of excitation pulse length (10 ns from OPO vs 4 ms from TTL-controlled CW laser diode) and pump wavelength (980 nm for UC and 520 nm for Stokes emission) on the RET behavior of lanthanide-doped core-shell donor particles, we have prepared differential rate equation (DRE) Yb-Er UC model (based on^37,41–48^, details in SI—Tables S7,S8). These DRE equations were fed with rate parameters taken from ref. ^41^, which enables to a simulation of luminescence kinetics under short (around 10 ns) and high energy peak pulsed excitation.

## Supplementary information

supplementary file
