# Supplementary material for: Upconversion FRET quantitation: the role of donor photoexcitation mode and compositional architecture on the decay and intensity based responses
Source: Light Sci Appl. 2022 Aug 19;11:256. doi: 10.1038/s41377-022-00946-x (PMC9391450; doi:10.1038/s41377-022-00946-x)
Supplement: Supplementary file 1 — supplementary file [file 41377_2022_946_MOESM1_ESM.docx]

Supplementary Information for

Upconversion FRET quantitation: the role of donor

photoexcitation mode and compositional architecture

on the decay and intensity based responses

Agata M. Kotulska^a^, Aleksandra Pilch-Wróbel^a^, Satu Lahtinen^b^,
Tero Soukka^*b^, Artur Bednarkiewicz^*a^

^a^ Division of Biomedical Physicochemistry, Institute of Low Temperature and Structure Research, PAN, ul. Okolna 2, Wrocław 50-422, Poland

^b^ Department of Life Technologies/Biotechnology, University of Turku, Kiinamyllynkatu 10, 20520 Turku, Finland

*Corresponding authors e-mails:

Tero Soukka: tejoso@utu.fi

Artur Bednarkiewicz: [a.bednarkiewicz@intibs.pl](mailto:a.bednarkiewicz@intibs.pl)

[I. Nomenclature - 2 -](#_Toc108214161)

[II. Introduction for calculating resonance energy transfer efficiency - 2 -](#_Toc108214162)

[II.A. LRET efficiency calculations - 2 -](#_Toc108214163)

[II.B. Theoretical emission and absorption for donor and acceptor - 4 -](#_Toc108214164)

[III. Materials and methods - 5 -](#_Toc108214165)

[Materials - 5 -](#_Toc108214166)

[Preparation of precursor - 5 -](#_Toc108214167)

[Synthesis of upconverting nanoparticles - 5 -](#_Toc108214168)

[Removal the oleic acid - 6 -](#_Toc108214169)

[Characterization of synthesized nanoparticles - 6 -](#_Toc108214170)

[Rose Bengal dye titration - 7 -](#_Toc108214171)

[IV. Spectroscopic Measurements - 8 -](#_Toc108214172)

[V. The procedure of fitting the luminescence lifetimes - 11 -](#_Toc108214173)

[VI. Mathematical methods (kinetic modelling) - 24 -](#_Toc108214174)

[VI.A. Equations and parameters for kinetics modeling - 25 -](#_Toc108214175)

[VI.B. Influence of power and concentration on donor luminescence lifetimes - 31 -](#_Toc108214176)

[VII. References - 33 -](#_Toc108214177)

# Nomenclature

Following the majority of studies, we use the term RET to describe the most general mechanism of resonant energy transfer between excited donor and ground state acceptor. In this sense, the RET term does not distinguish between various mechanisms leading to e.g. intersystem crossing, sensitized up conversion, or conventional Stokes excitation. Neither does it consider any specific types or features of *D* or *A* species, which can be individual molecule, individual quantum dot donors, or multiple donor ion doped nanoparticles. The term FRET is used in its conventional sense to specifically describe RET involving conventional molecular *D* and *A* species, while the LRET will be used to discuss RET involving luminescent lanthanide ions as donors, either from molecular lanthanide complexes or lanthanide doped nanoparticles. To consider the up conversion as the photoexcitation mechanism proceeding RET between excited states of multiple lanthanide ions within single donor nanoparticles and multiple acceptor molecules anchored to the surface of such donor nanoparticle, we adopt the term UC-LRET. In particular, the UC-LRET with upconverting nanoparticles involves discussion of the energy-transfer upconversion (ETU), energy migration (EM), and back energy transfer (BET). All of them could influence the excitation of the upconverted, resonant state of the donor-activator ions in the UCNPs and potential rapid ‘recharging’ of the actual donor ions relaxed upon UC-LRET. In some cases, the effect of sensitization is common using antenna dye-sensitized downshifting or sensitizer-ETU-based upconverting lanthanide donors. In both cases, the QY of the donor related to RET is the internal QY of the donor that does not include the efficiency of the sensitization path. Thus, the existence of energy transfer steps in the donor excitation pathway does not alone fundamentally differentiate Eu-chelate and UCNP donor based RET ^1^. However, with Ln^3+^ as a donor, photoexcitation pathway is far more complex, and each UCNP is composed of thousands or tens of thousands of actual Er^3+^ donor ions and sensitizing Yb^3+^ . The features that make the UC-LRET were discussed here, to be so unique in behavior.

# Introduction for calculating resonance energy transfer efficiency

## LRET efficiency calculations

Förster radius (at which LRET parameters such as decay rates are in equilibrium
($k_{FRET}=k_{D}^{R}+k_{D}^{NR}=\tau_{D}^{-1}$) and efficiency of LRET (η_LRET_) is equal 50%) (equation 5.4 in ^2^):

| $R_{0}=\left( \frac{9\left( \ln10 \right)\kappa^{2}\Phi_{D}}{128\pi^{5}N_{A}n^{4}}J \right)^{1/6}$ | Eq. S1 |
| --- | --- |

Where $\kappa^{2}$ is the orientation factor (for most cases $\kappa^{2}=2/3$ for dynamic and isotropic distribution of *D* against *A*, this parameter takes a value between 0 and 4), $\Phi_{D}$ is donor quantum yield (takes a value between 0 and 1), $N_{A}$ is Avogadro’s number, $n$ is refractive index of the surrounding medium.

Assume $N_{A}=6.0221415\times{10}^{23}$ mol^-1^, $ln(10)=2.302585093$, $\pi=3.141592654$ (page 26 in ^2^) – if we consider all constants ($9(\ln\left( 10 \right) )/(128\pi^{5} )=8.79\times{10}^{-28} mol$ [equation 5.10 in ^2^]), Förster radius can be described as [equation 5.11 in ^2^]

| $R_{0}=\left( 8.79\times{10}^{-5}\cdot\Phi_{D}J_{\lambda}n^{-4}\kappa^{2} \right)^{1/6} [\left( molM^{-1}cm^{-1}nm^{4} \right)^{1/6}]$ | Eq. S2 |
| --- | --- |

Calculation of Förster radius with equation (3.3a and 5.12 in ^2^), taking into account units of spectral overlap (J) $M^{-1} cm^{-1} nm^{4}= {10}^{17} nm^{6} mol^{-1}$

| $R_{0} [nm] =0.02108\cdot\left( \kappa^{2}\cdot\Phi_{D}\cdot n^{-4}\cdot J \right)^{1/6}$ | Eq. S3 |
| --- | --- |

According to Lambert-Beer law from measured absorbance spectrum can be calculated molar extinction coefficient ($c$ is concentration of sample, $l$ is light path length – 1 cm for standard cuvette) (equation 5.30 in ^2^):

| $A\left( \lambda\right)=\varepsilon\left( \lambda\right)\cdot c\cdot l\Longrightarrow\varepsilon\left( \lambda\right)=\frac{A\left( \lambda\right)}{c\cdot l}$ | Eq. S4 |
| --- | --- |

Integral overlap defined in wavelength (λ) or wavenumber ($\tilde{\nu}$) scale is equal (equation 5.5 and 12.3 in ^2^) and additionally could be described in terms of normalized intensity under :

| $J=\frac{\int F_{D}\left( \lambda\right)\varepsilon_{A}\left( \lambda\right)\lambda^{4}d\lambda}{\int F_{D}\left( \lambda\right)d\lambda}=\int f_{D}(\lambda)\varepsilon_{A}(\lambda)\lambda^{4}d\lambda=\int f_{D}\left( \tilde{\nu} \right)\varepsilon_{A}\left( \tilde{\nu} \right)d\frac{\tilde{\nu}}{\tilde{\nu}^{4}}$ | Eq. S5 |
| --- | --- |

where $\varepsilon_{A}$ is a spectrum of acceptor molar absorptivity (or extinction coefficient), $I_{D}$ is donor emission spectrum normalized to unity ((M^-1^ cm^-1^) – liter per mole per centimeter).

LRET mechanism is based on dipole – dipole interaction through energy transfer rate $k_{FRET}$ (equation 12.1), $\tau_{D}^{-1}=k_{r}+k_{nr}=k_{0}$ is a sum of radiative and nonradiative rates for donor, $r$ is distance between donor and acceptor (equation 12.1 in ^2^):

| $k_{FRET}=\frac{1}{\tau_{D}}\left( \frac{R_{0}}{r} \right)^{6}$ | Eq. S6 |
| --- | --- |

$\varepsilon_{A}(\lambda)$ is normalized molar extinction coefficient of the acceptor in M^-1^ cm^-1^. Overlap-integral unit (OLI) can be changed according to unit conversion equation ${10}^{14} mol^{-1} dm^{3} cm^{-1} nm^{4} = {10}^{-14} mol^{-1} dm^{3} cm^{3}$ (page 28 in ^2^). LRET efficiency between *D* and *A* can be described as below (100 % at $r<0.5\cdot R_{0}$, 0 % at r$>1.5\cdot R_{0}$)

| $\eta=\frac{k_{FRET}}{k_{0}+k_{FRET}}= \frac{R_{0}^{6}}{R_{0}^{6}+r_{DA}^{6}}=1-\frac{\tau_{DA}}{\tau_{D}}=1-\frac{I_{DA}}{I_{D}}$ | Eq. S7 |
| --- | --- |

Where $R_{0}$ is Förster distance (at which for studied $D$ – $A$ pair efficiency equals 50%),
$r_{DA}$ – distance between $D$ and $A$, $\tau_{D}$, $\tau_{DA}$ and $I_{D}$, $I_{DA}$ are luminescence lifetimes and intensities of $D$ without or with presence of $A$. Additionally when we calculate Förster distance for this pair this would be equal $2.001\pm0.24 (nm).$ ^3^

Because we evaluate luminescence rise and decay times separately (for one of three photoexcitation cases), the **Eq. S8** is used to compare risetime of pure donor $\tau_{R,D}$ with the rise time of donor in the presence of acceptor $\tau_{\boldsymbol{R},DA}$ (the RET efficiency indicated as $\eta(\tau_{\boldsymbol{R}})$).

| $\eta\left( \tau_{\boldsymbol{R}} \right)=1-\frac{\tau_{\boldsymbol{R}, DA}}{\tau_{\boldsymbol{R}, D}}$ | Eq. S8 |
| --- | --- |

Similarly, the RET efficiency based on luminescence decays (**Eq. S9**) were calculated with the same equation as the luminescence decay for the luminescence lifetime of donor ($\tau_{\boldsymbol{D},D})$and the luminescence lifetime of donor in the presence of acceptor $\tau_{\boldsymbol{D},DA}$ (the RET efficiency indicated as $\eta(\tau_{\boldsymbol{D}})$).

| $\eta\left( \tau_{\boldsymbol{D}} \right)= 1-\frac{\tau_{\boldsymbol{D},DA}}{\tau_{\boldsymbol{D},D}}$ | Eq. S9 |
| --- | --- |

## Theoretical emission and absorption for donor and acceptor

To estimate the spectral overlap between *D* and *A* required for the LRET mechanism, we provided a theoretical calculation based on the absorption cross-section of Er^3+^, the literature value of molar extinction for Rose Bengal (RB) dye (that allow to estimate the intensity of the sensitized acceptor emission), and also the emission spectrum. Only ^2^H_11/2_ (at 520 nm) and ^4^S_3/2_ (at 540 nm) emission overlaps with the absorption bands of RB. Energy levels for Er^3+^ lanthanides ions are shown in **Figure 1** and were analyzed theoretically with Judd Ofelt theory and differential rate equation, and experimentally with emission spectra. Molar extinction coefficient (M^-1^ cm^-1^) of RB gives information on how dye absorbs light at each wavelength. The spectral overlap for Er^3+^ ion emission and RB absorption spectrum fulfills the requirements for RET mechanism. In our system, we photoexcite Yb^3+^ ions with 980 nm laser wavelength, which is far from absorption of the acceptor (RB) in the studied resonance energy transfer scheme, so we can disregard residual *A* photoexcitation and focus on $D$-$A$ RET mechanism.

**Figure S1:** **Theoretical absorption cross-section of Er^3+^ ion from ground state** calculated based on Judd-Ofelt theory ^4^ and theoretical extinction coefficient and emission of RB dye ^5^.

# Materials and methods

### Materials

Yttrium oxide (99.99 %), ytterbium oxide (99.99 %), and erbium oxide (99.99 %) were purchased from Alfa Aesar. Dry DMF (99.8 %), nitrosonium tetrafluoroborate (95 %), Rose Bengal (95 % dye content), oleic acid (OA, 90 %), and 1-octadecene (ODE, 90 %) were purchased from Sigma Aldrich. Acetic acid (99.5 - 99.9 %), ethanol (96 %), *n*-hexane (95 %), methanol (99.8 %), ammonium fluoride (98 %), toluene (99,5 %), acetonitrile and sodium hydroxide (98.8 %) were purchased from Avantor Performance Materials S.A. (Poland). All chemical reagents were used without further purification. The materials were synthesized according to the method described in previous publications ^6,7^.

### Preparation of precursor

The precursor was generally prepared by mixing stoichiometric amounts of lanthanide oxides (Y_2_O_3_, Yb_2_O_3_, Er_2_O_3_—1 × 10^−3^ M) with 50 % aqueous acetic acid and heating the mixture to 200 °C for 120 min under pressure. The final precursor was obtained by evaporating residual acid and water in a rotary evaporator, and further drying at 130 °C for 12 h.

### Synthesis of upconverting nanoparticles

*Preparation of Core and Core@Shell Material*: In a typical synthesis, the given amounts (2 × 10^−3^ mol Ln^3+^) of (CH_3_COO)_3_Ln precursors were added to the three-neck flask with OA (12 cm^3^) and ODE (30 cm^3^). The solution was stirred under a nitrogen atmosphere and heated slowly to 140 °C, followed by degassing under a vacuum for 30 min to remove oxygen and water. After evaporation of residual water, the nitrogen atmosphere was maintained during the synthesis. Then, the reaction temperature was decreased to 50 °C, and during this time, solutions of ammonium fluoride and sodium hydroxide dissolved in methanol were added. The reaction mixture was stirred for 30 min at 50 °C. Then, the temperature was increased to 80 °C, and the mixture was kept at this temperature for 30 min to evaporate methanol. After that, the reaction temperature was increased quickly to 300 °C and kept at this temperature for 60 min under a nitrogen atmosphere. After the UCNPs formation, the mixture was allowed to cool to room temperature. The UCNPs were precipitated by the addition of ethanol and isolated by centrifugation at 10 000 rpm for 10 minutes. For purification, the resulting pellet was dispersed in a minimal amount of *n*-hexane and again precipitated with excess ethanol. The final product was isolated by centrifugation at 14 000 rpm for 10 min and dispersed in 12 cm^3^ *n*-hexane. For the synthesis of core @ shell UCNPs, the following changes in the reaction procedure were introduced. After initial shell precursor mixing and dissolving in OA and ODE at 140 °C, the mixture was cooled to 80 °C, and the solution of core UCNPs was added. The reaction solution was maintained at 80 °C to remove *n*-hexane. Then, the temperature was lowered to 50 °C, and methanolic solutions of ammonium fluoride (8 × 10^−3^ mol) and sodium hydroxide (5 × 10^−3^ mol) were added. The next steps of the synthesis were analogous to the previously described procedure used for core synthesis. Finally, the UCNPs were precipitated and centrifuged using the same procedure mentioned above. The final product stabilized with OA ligands was dispersed in 5 cm^3^ of chloroform (CHCl_3_).

### Removal the oleic acid

To remove the oleic acid ligands from the surface, the 8 mg of UCNPs was transferred to the Eppendorf tube, then after the centrifugation (30 min, 16900 g), the 500 µl of hexane was added. The mixture was sonicated as long as the pellet has been suspended. After that, the 400 µl of acetonitrile and 100 µl of 0.16 M NOBF_4_ in acetonitrile were added to the tube, and the solution was mixed until the particles have transferred from the upper hexane layer to the lower acetonitrile layer. In the next step, the hexane layer has been removed carefully with a pipette. Finally, the particles were precipitated by adding the 500 µl of toluene, vortexed, centrifuged (30 min, 16900 g), and after removal of the supernatant, re-dispersed in 100 µl of dry DMF.

### Characterization of synthesized nanoparticles

Powder diffraction data (**Figure S2**) of the synthesized UCNPs were collected on an X’Pert PRO X-ray diffractometer with a PIXcel ultrafast line detector, a focusing mirror, and Soller slits for Cu Kα radiation. The measurements were done in a Bragg–Brentano geometry in the 10–120 2Θ range. The XRD patterns were assigned using the Joint Committee on Powder Diffraction Standards (JCPDS) database.

Morphology and microstructure were investigated with TEM-EDX (double CS-corrected Titan3 G2 60-300 transmission electron microscope by FEI Company). Severe NP degradation of the NPs occurred under fast under the electron beam. TEM studies of core and core-shell UCNPs show the nanoparticle size (diameter) distribution.


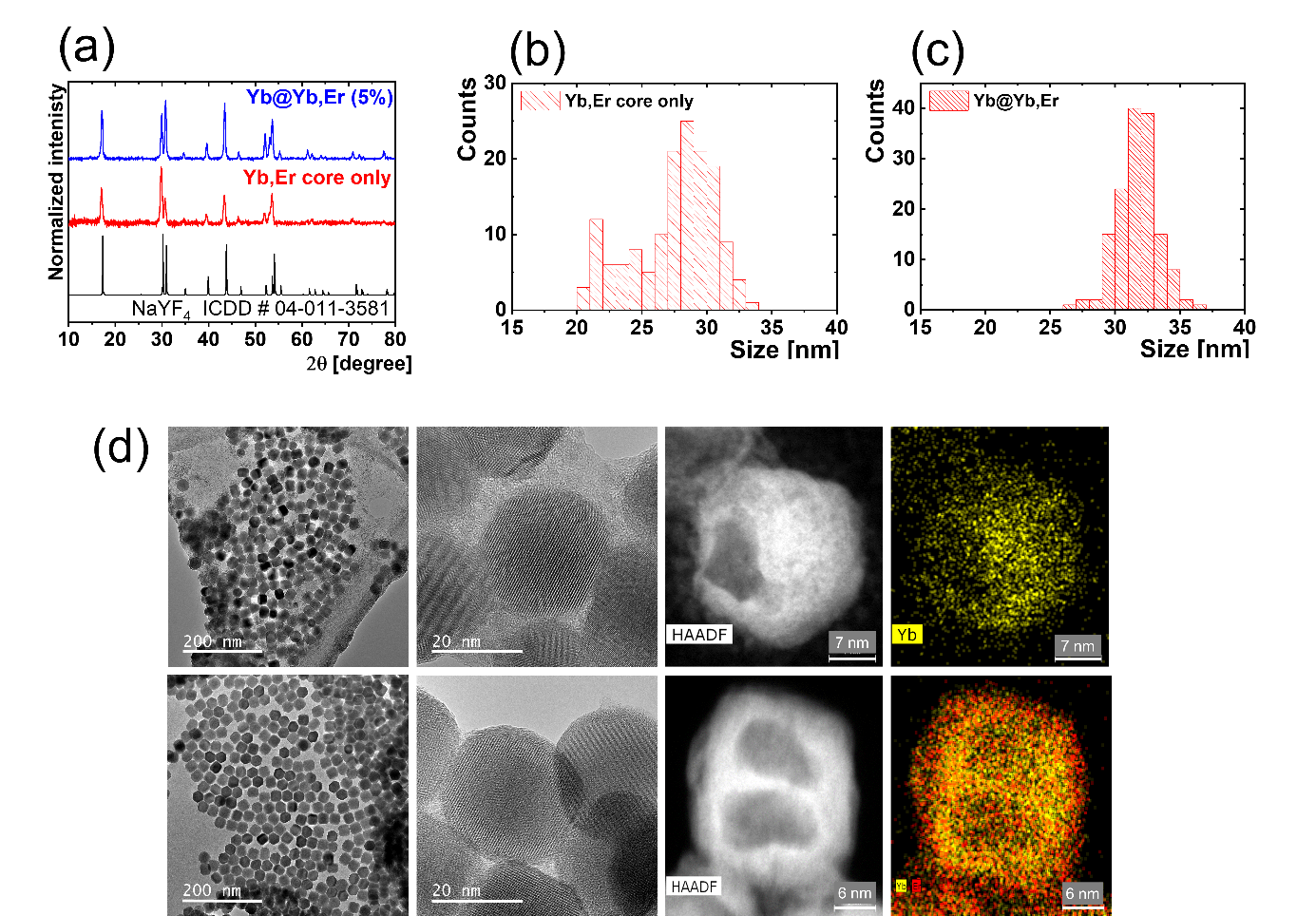


**Figure S2:** XRD, TEM, EDS, and size distribution of the samples under studies.

### Rose Bengal dye titration

Both the *D* and *A* have to be conjugated to other molecules (e.g. antibodies, DNA fragments, etc.) to enable modulation of their distance by biospecific reaction molecules and use in biosensing applications. However, performing experiments involving complexes formed by biospecific binding reactions such as DNP-Ab: Ag-A, where DNP-Ab means donor NP conjugated antibody (Ab) and Ag-A acceptor molecule (A) conjugated antigen (Ag), is complex, tedious, and expensive, and also prone to experimental variation. Thus a simplified DNP: A sensing approach based on anchoring acceptor directly on donor nanoparticle was selected. By coordinating the dye directly to the surface, we are able to minimize the *D*– *A* distances and see the maximal response available upon LRET using the two structurally and compositionally different UCNPs as donors. It is true, that in the practical bioassays, the surface chemistry and conjugation will inevitably render the distances longer. Still, the study based on the direct binding of the dye on the surface, however, minimizes all the uncertainly related to conjugation chemistry and biological interactions resulting in variation in the distances and binding efficiencies.

Due to the fact that LRET and reabsorption of donor emission both give a similar spectral response, distinguishing between LRET and the reabsorption process is not a trivial task. In both cases, the sensitized emission of the acceptor dye will be observed upon excitation of the donor. In the proposed surface blocking experiment (**Figure S3**), we examine the distance dependency of LRET. The idea of it was to measure the intensity of the sensitized acceptor emission in two different cases: (1) the acceptor was attached directly to the surface, i.e. the distance between *D* and *A* is minimized, resulting in the occurrence of non-radiative, resonance energy transfer from the donor to acceptor; (2) the UCNP surface was blocked by using phosphate buffer. Thus, two dye titration series were performed for each particle batch: one with and the other without phosphate buffer. First, 0.4 mg of UCNP’s was added to each of the samples to the Eppendorf tubes of dry DMF. Then aqueous phosphate buffer was added in 1 µl to the one series of the samples. After 15 minutes of incubation, the RB acceptor dye was added and dissolved in dry DMF in six concentrations (0, 2.9, 9.8, 29.5, 98.3, and 294.8 µM).

The phosphates from the buffer in the second step bind to the surface, promoting negative charge and repulsion towards the acceptor dye, and leaving no space for attachment of the dye. In this case, the acceptor dye is only in the surrounding solution and not immediately on the surface of the UCNPs. In solution, the dye provides the conditions for reabsorption of the donor emission, but since the distance between donor and acceptor is on average much beyond the Förster distance, the possibility of LRET is minimized. The results of our experiments indicated that the sensitized acceptor emission is observed only when the acceptor dye is attached directly to the surface. When the surface is blocked with phosphate, the emission of the acceptor dye is not observed at all with experimental conditions used (i.e. acquisition time) used. Our results allow to conclude that the observed sensitized emission of the acceptor is excited non-radiatively, i.e. through LRET from the Er^3+^ ions to the RB molecules.


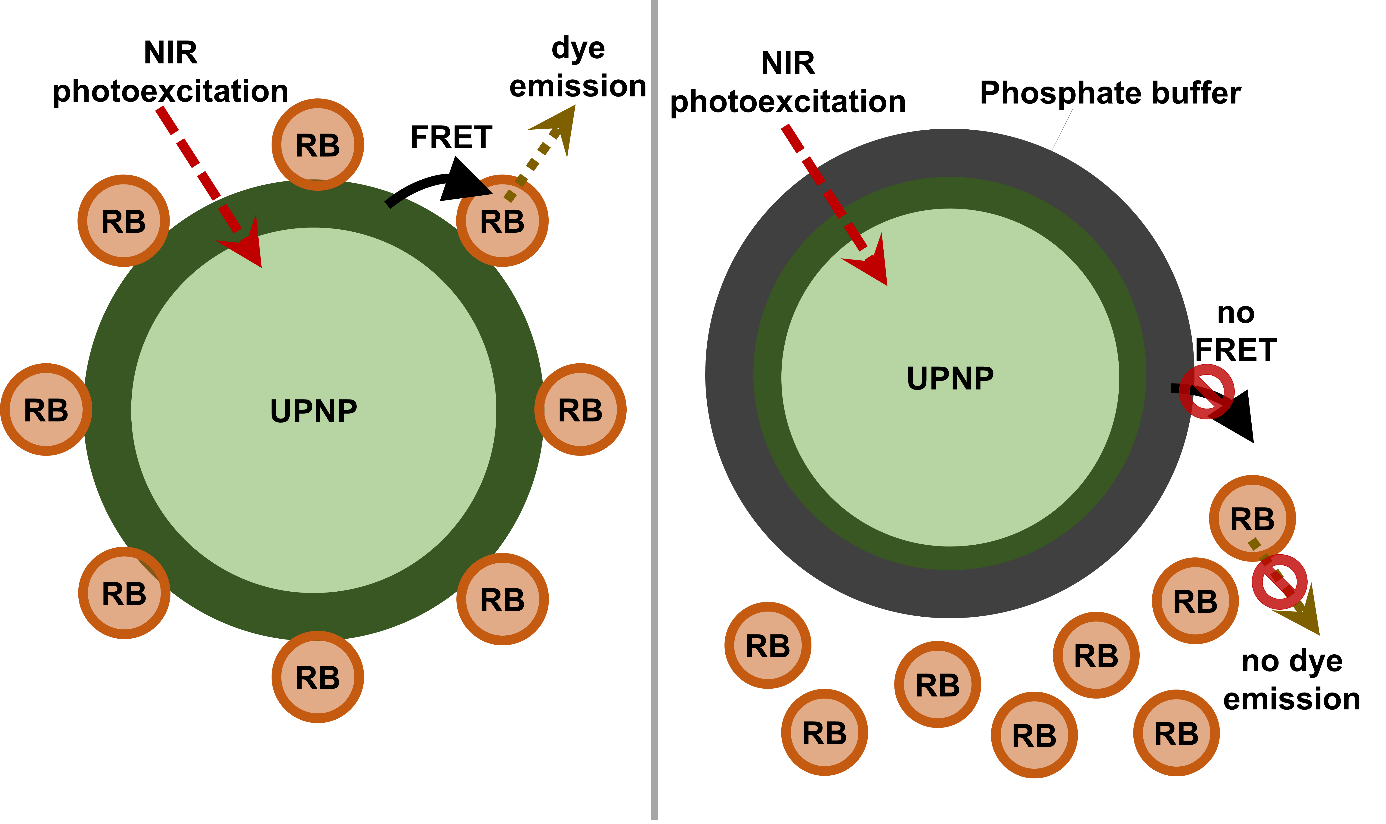


**Figure S3:** Scheme of LRET occurrence experiment using UCNP donor and RB acceptor dye both without (left) and (right) phosphate blocking.^3^

# Spectroscopic Measurements

Typically, to get bright up-conversion emission, continuous wave (CW) laser diode excitation is used to pump the sensitizer ions, which then continuously up-convert their energy to the higher levels of the activator ions. After that, the excited activator ions, which act concurrently as donor ions to the acceptor, deliver the excited state energy via LRET to the acceptor molecules. In pulsed mode, activator emission is typically achieved with powerful pulsed lasers (e.g., OPO, Ti: sapphire, dye lasers, etc.) or with a TTL modulated continuous wave laser diodes (LD), which are in the medium-low CW power range. While the pulsed laser sources are straightforward and a natural approach for luminescence lifetime measurements (and RET quantification), such costly and large footprint lasers are not well suited for RET based assays aimed for field use or point-of-care applications. Therefore, we decided to additionally study and compare the performance of our UC-LRET assay under such TTL modulated CW laser diode. LDs are more compact and robust light sources, with an accessible power supply and control, and thus is a more cost-effective solution for point-of-care miniaturized reading devices. However, due to the complex nature of the sensitized energy up-conversion processes, relatively long pulses obtained by TTL modulated low/medium power CW laser diodes may not be suitable for LRET studies in luminescence kinetic mode.

Luminescence decay lifetimes were measured with three different kinds of pulse excitation: short pulse excitation was performed by tunable laser OPOLLETE 355 LD Optical Parametric Oscillator (OPO) for 980 nm excitation wavelength with median pulse energy 0.093 mJ (**OPO980**), and for 520 nm with measured median pulse energy 0.107 mJ (**OPO520**), and long 10 ms pulse excitation was performed by TTL controlled infrared CW diode laser MDL-F-980-10 W with a median value of pulse energy 0.145 mJ (**TTL980**) (**Table S. 1**). Measurements of pulse energies were performed with ES120C Pyroelectric Energy Sensor (Thorlabs). Laser pulses for TTL980 photoexcitation mode were the longest, and had the highest energy value, while the photoexcitation with OPO980 and OPO520 had 100 times shorter pulse and around 35 and 50 % lower average energy, but much larger momentary pulse intensity than in TTL980 (**Table S1**).

Table S1: Experimental parameters of photoexcitation three types of laser pulses.

| Laser pulse | TTL980 | OPO980 | OPO520 |
| --- | --- | --- | --- |
| Energy | 0.141 mJ | 0.093 mJ | 0.070 mJ |
| Pulse length | 4 ms | 5-7 ns | 5-7 ns |
| Repetition of pulse | 10 ms (100 Hz) | 50 ms (20 Hz) | 50 ms (20 Hz) |

Donor and acceptor wavelength specific luminescence kinetics was recorded using a photomultiplier tube module (bandwidth 80 MHz, averaging of 1000 pulses) and optical filters characterized in Figure S5. Emission spectra intensity measurements were carried out with CW laser diode (980 nm, ~5 W) excitation and OceanOptics HR4000CG spectrometer (5 s of integration time) readout.

All the luminescence measurements were carried out for colloidal samples in cuvettes in a homebuilt setup (**Figure S4**), in homemade quartz cuvettes (3 mm internal diameter, 1.5 mm light path from the center) with a 90^o^ light collection configuration. Following equation $T \left[ \% \right]=100\cdot{10}^{-\varepsilon lc}$, and assuming 90.400 (cm^-1^ M^-1^]) at 560 nm, 0.15 cm optical length (from the center of quartz cuvette to the edge where the readout is made), as well as assuming the 10-fold diluted sample were used in the experiments, we obtain with the highest RB concentration a theoretical value of transmission equal 40 %, which in consequence may modify the shape of emission spectrum that originates from within the center of the cuvette. Indeed, a slight, apparent spectral shift of the RB emission observed by us for the rising concentration of RB should be rationalized by the spectral overlap between RB emission and its absorption ^8^. The molar absorption coefficient of RB also drops at wavelengths below 550 nm and thus the effect of RB absorption to donor emission is less than the theoretical value estimated. Additionally, as shown in RB emission is composed of two emission bands. In the absorption spectra, there are also bands at 570 nm and 620 nm, which likely originate from monomer (longer wavelength) and dimer (shorter wavelength) forms of the RB dye, respectively ^9^. Monomeric and dimeric fractions do not influence quantum yields for RB, and RET transmission is influenced by monomers. ^10^


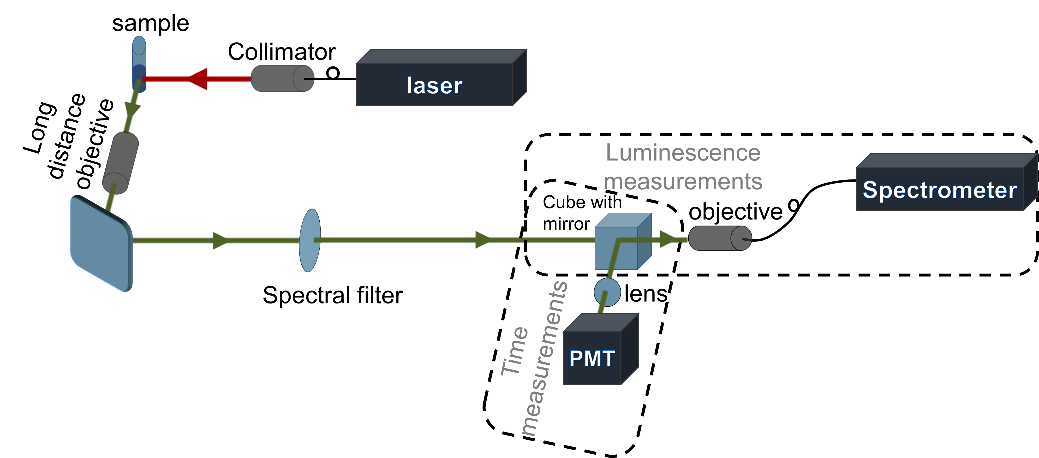


**Figure S4: Experimental setup scheme** with laser, collimator, a place for cuvette with the sample, long distance objective with two different arms for (a) luminescence and (b) time measurements. Characterization of laser pulse used to compare photoexcitation scheme is presented in **Table S1**. Spectral properties of optical filters used in the experimental setup are given in **Figure S5**.

Both the ^2^H_11/2_ and ^4^S_3/2_ energy levels are suitable for RET to RB. The ^2^H_11/2_ stays in thermal equilibrium with the ^4^S_3/2_ level governed by Boltzmann law. Moreover, the ^2^H_11/2_ level is rapidly and non-radiatively populating the ^4^S_3/2_ level. Therefore, the green Er^3+^ emission is often described as the ^2^H_11/2_ + ^4^S_3/2_*.* It is simple to extract the emission bands of ^2^H_11/2_ and ^4^S_3/2_ from UC spectra (Fig.2), but to measure luminescence lifetimes, we simplified the setup and measured only ^4^S_3/2_ (green) and ^4^F_9/2_ (red) emissions (**Figure S4**). To measure the luminescence emission lifetimes of the ^4^S_3/2_ and ^4^F_9/2_,we used two different bandpass filters placed in front of a PMT detector. The FF01-660/30 band-pass filter (transmission between 637 – 681 nm) was used for Er^3+^ ions ^4^F_9/2_ level and FB543.5-10 band-pass filter (transmission between 532 – 557 nm) was used for Er^3+^ ions for ^4^S_3/2_ level. The transmission curves of both filters are presented in Figure S5a. In addition, FF01-790/SP short-pass filter (790 nm cut-off wavelength) was used to exclude the laser pulse line at 980 nm. A FELH0550 (Thorlabs) filter was used to cut-off the 520 nm laser excitation in OPO520 experiments. These two filters were used in combination with either 980 nm (matching ^2^F_7/2_ → ^2^F_5/2_ of Yb^3+^, indirect Er^3+^ excitation through ETU) or 520 nm (matching ^4^I_9/2_ → ^2^J_11/2_, direct Er^3+^ ions excitation) excitation lasers. The signal from the PMT photodetector was averaged (1000 pulses) using a digital oscilloscope synchronized to the falling (for OPO520 and OPO 980) or rising (TTL980) edge of the laser pulse.


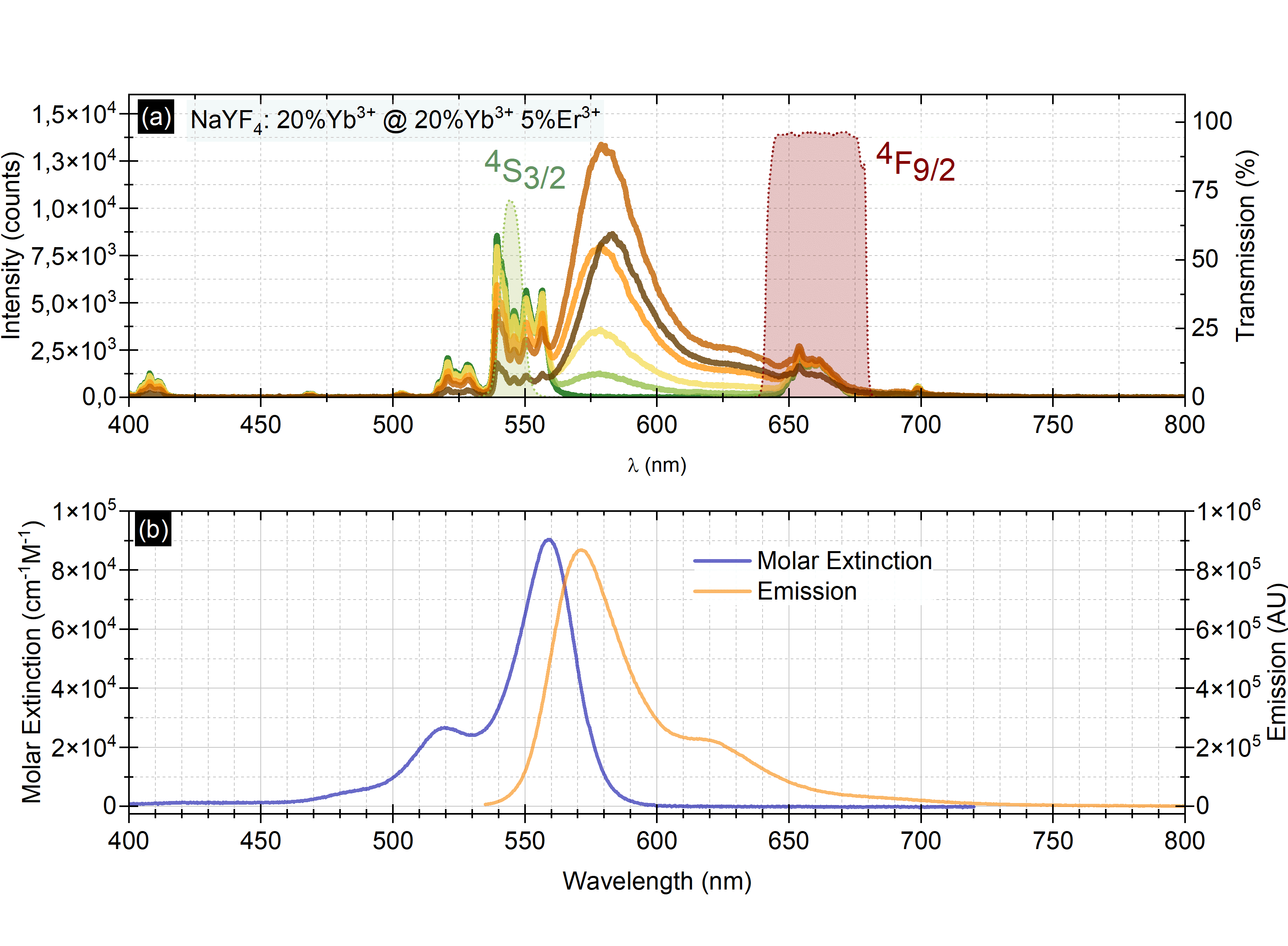


**Figure S5:** Transmission spectra of the band pass emission filters and spectral characteristics of the UC-LRET pair. (a) Characteristics of used band pass emission filters used for the luminescence spectra measurements FF01-660/30 band-pass filter (i.e. center wavelength 660 nm and 20 nm width at half-height of the transmission) for Er^3+^ ions ^4^F_3/2_ level, FB543.5 -10 for ^4^S_3/2_. (b) Molar extinction coefficient and emission spectrum of RB.

# The procedure of fitting the luminescence lifetimes

Depending on the photoexcitation scheme, we proposed and used three different methods to quantify the kinetic profiles of Er^3+^ emission, which are presented in **Table S2**.

As a supplementation of information from the main article (in **Figure 3** and **Figure 4**), we presented all (not only the leading and most significant changes) for luminescence kinetics and lifetimes for the ^4^S_3/2_ and ^4^F_9/2_ levels of Er^3+^ are shown in **Figures S6** and **S9**, and additionally supplemented it with residual of luminescence lifetime fitting. We present additionally emission of ^4^F_9/2_ for three types of photoexcitation, as supplementary for presented only in article emission under short indirect photoexcitation (OPO980), which indicate the most significant changes.

**Table S2:** The equations used to fit experimental data, each row represented the most simple description for the laser photoexcitation scheme. The A_1_, A_2_, A_3_ are amplitudes for luminescence lifetimes (τ) to fit with exponential functions. The n_i_ (i = 1, 2, 3) indicates the population of the three levels of Er^3+^ ions, β is an emission branching ratio from 3 → 1; W_1_ and W_2_ are rates of building and decaying the population of the emitting level.

| Laser type | Analyzed part of a lifetime | Model | Equation |
| --- | --- | --- | --- |
| OPO520 | Decay time | triple exponential lifetime | $\sum_{i} A_{i}\cdot\exp\left( -\frac{t}{\tau_{i}} \right)=A_{1}\cdot e^{\left( \frac{-t}{\tau_{1}} \right)}+A_{2}\cdot e^{\left( \frac{-t}{\tau_{2}} \right)}+A_{3}e^{\left( \frac{-t}{\tau_{3}} \right)}$ |
|  |  | analysis with average | $\tau_{Av}=\frac{\sum_{i} A_{i}\cdot\tau_{i}}{\sum_{i} A_{i}}=\frac{A_{1}\cdot e^{\left( \frac{-t}{\tau_{1}} \right)}+A_{2}\cdot e^{\left( \frac{-t}{\tau_{2}} \right)}+A_{3}e^{\left( \frac{-t}{\tau_{3}} \right)}}{A_{1}+A_{2}+A_{3}}$ |
| OPO980 | Rise and decay time | differential rate model | $W_{1}=1/\tau_{R}$  $W_{2} =1/\tau_{D}$  $fun =$  $-I\cdot n_{1}+n_{2}\cdot W_{2}+ \left( 1-\beta\right)\cdot n_{3}\cdot W_{1}$  $\beta\cdot n_{3}\cdot W_{1} - n_{2}\cdot W_{2}$  $I\cdot n_{1} - (n_{3}\cdot W_{1})$ |
| TTL980 | Rise time | Mono exponential | $I_{0}+1-A_{1}\cdot e^{\left( \frac{-\left( t - t_{0} \right)}{\tau_{R}} \right)}$ |
|  | Decay time | Double exponential | $I_{0}+A_{1}\cdot e^{\left( \frac{-\left( t-t_{0} \right)}{\tau_{1}} \right)}+A_{2}\cdot e^{\left( \frac{-\left( t-t_{0} \right)}{\tau_{2}} \right)}$ |

Luminescence lifetime values were presented in **Figure S8**,**S11** and **Table S3**, **S4**. Based on luminescence decay lifetime we estimated the efficiency of resonance energy transfer (RET) (**Figure S7** and **S10**). These kinetic data indicate that direct photoexcitation of Er^3+^ ions gives very efficient Resonance Energy Transfer, and some differences between two types of architectures can be observed, which owe most probably to differences in the number of ions. However, a higher absorption cross-section of Yb^3+^ gives higher intensity, and needs lower power density to photoexcite nanoparticles, enhances energy migration and energy storage cumulation, and may repump (“recharge”) Er^3+^ ions which hinder ultimate RET efficiency. Interestingly the highest changes in RET efficiency are visible for photoexcitation with a short pulse with 980 nm wavelength (OPO980). Long 980 nm photoexcitation pulse (TTL980) has minimal efficiency changes with dye concentration changes, which can be explained by recharging of Er^3+^ ions with energy transfer upconversion from Yb^3+^ ions due to longer laser pulses, cause greater participation of energy migration that can occur between Yb^3+^ ions. Surprisingly the most significant changes are visible for the rise time of ^4^F_9/2_, which cannot be directly related to the energy transfer from this level to the singlet state of Rose Bengal.

Similar conclusions about potentially misleading interpretation of rise- decay kinetics of UC in response to RET were recently drawn by Alyatkin et al. in Tm^3+^/ Yb^3+^ doped UC ^11^.


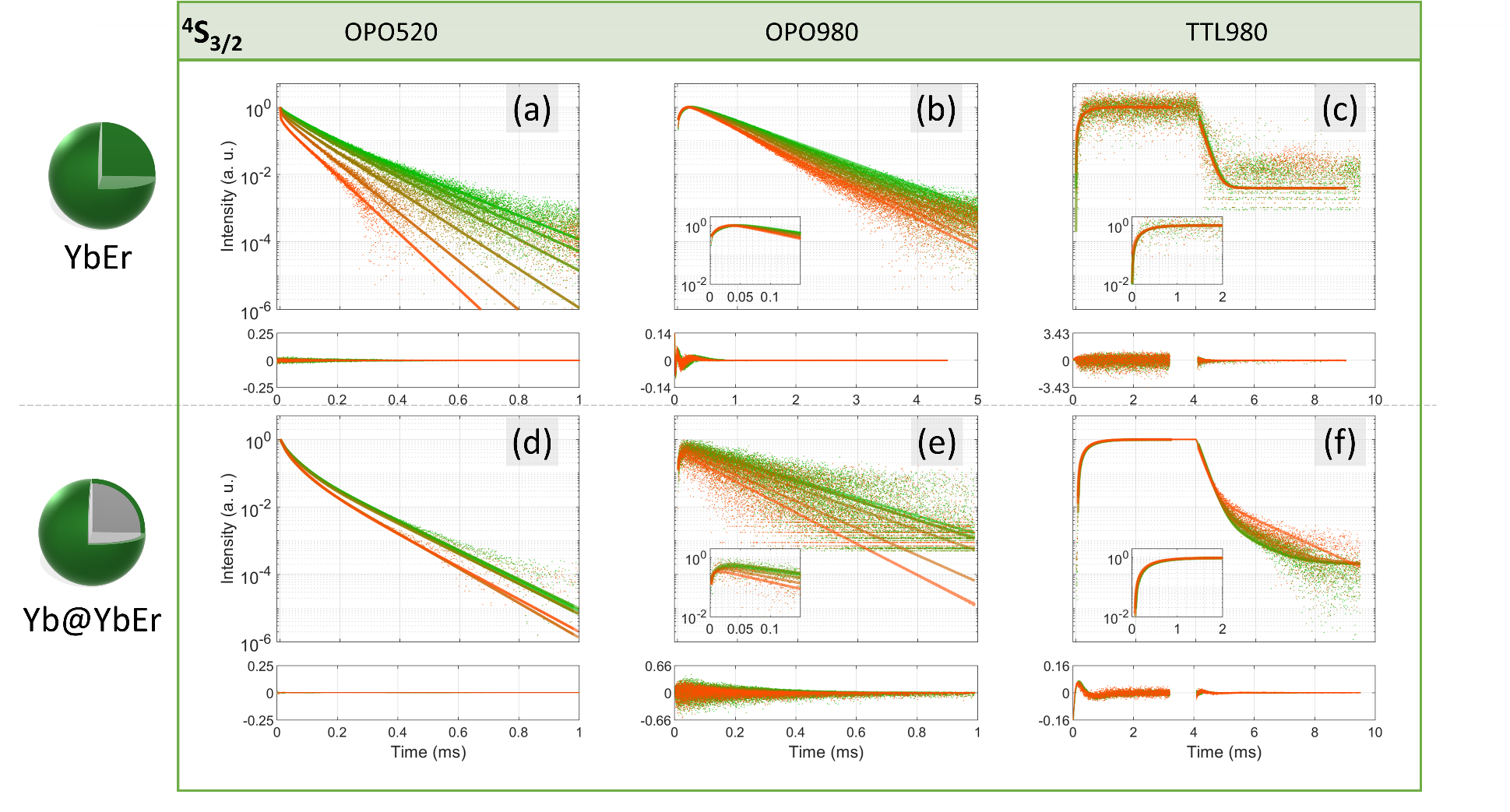


**Figure S6: Luminescence kinetics** from ^4^S_3/2_ energy level of Er^3+^ ions with fitting curves and residual of fits under each lifetime curve, for different photoexcitation mode – direct Er^3+^ ions photoexcitation with OPO520 (a, d), Yb^3+^ ions photoexcitation with OPO980 (b, e), and Yb^3+^ ions photoexcitation with longer pulse TTL980 (c, f). First row (a, b, c) shows results for core sample (NaYF_4_: 20% Yb^3+^ 2% Er^3+^), 2^nd^ row (d, e, f) shows results for core-shell sample (NaYF_4_: 20% Yb^3+^ @ NaYF_4_: 20% Yb^3+^ 5% Er^3+^).


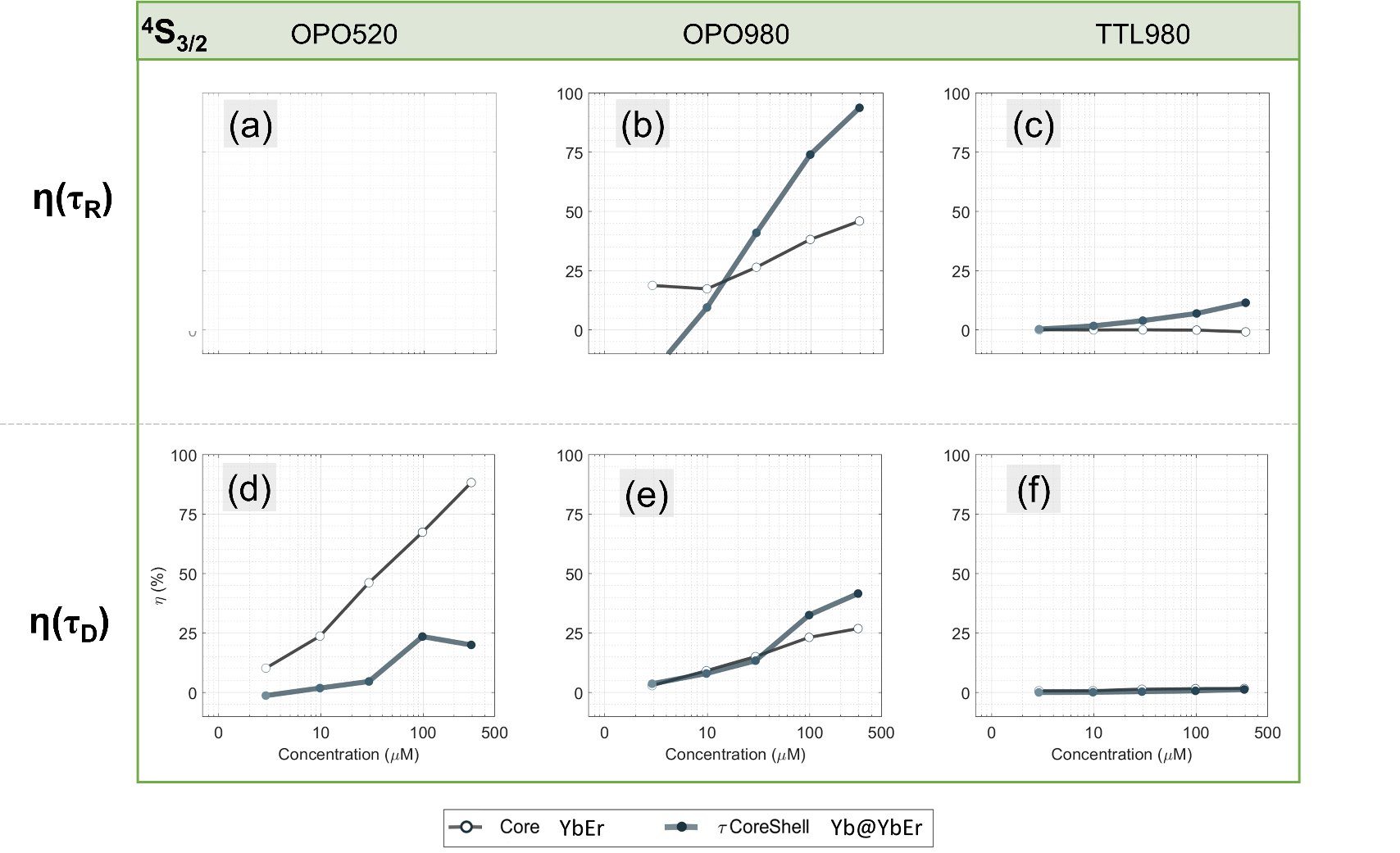


**Figure S7: Efficiencies of RET for ^4^S_3/2_** energy level of Er^3+^ ions. Efficiencies are based on luminescence kinetics of D with the absence of A versus D only kinetics. Either rise time (b, c) or averaged decay lifetime (d, e, f) for different photoexcitation mode were presented: direct Er^3+^ ions photoexcitation with OPO520 (a, d), Yb^3+^ ions photoexcitation with OPO980 (b, e), and Yb^3+^ ions photoexcitation with longer pulse TTL980 (c, f). Dark grey with filled dots shows results for the core sample (NaYF_4_: 20% Yb^3+^ 2% Er^3+^), and light grey with empty circles shows results for the core-shell sample (NaYF_4_: 20% Yb^3+^ @ NaYF_4_: 20% Yb^3+^ 5% Er^3+^). No data are provided in (a) because no rise times were observed under Stokes excitation (at 520 nm).


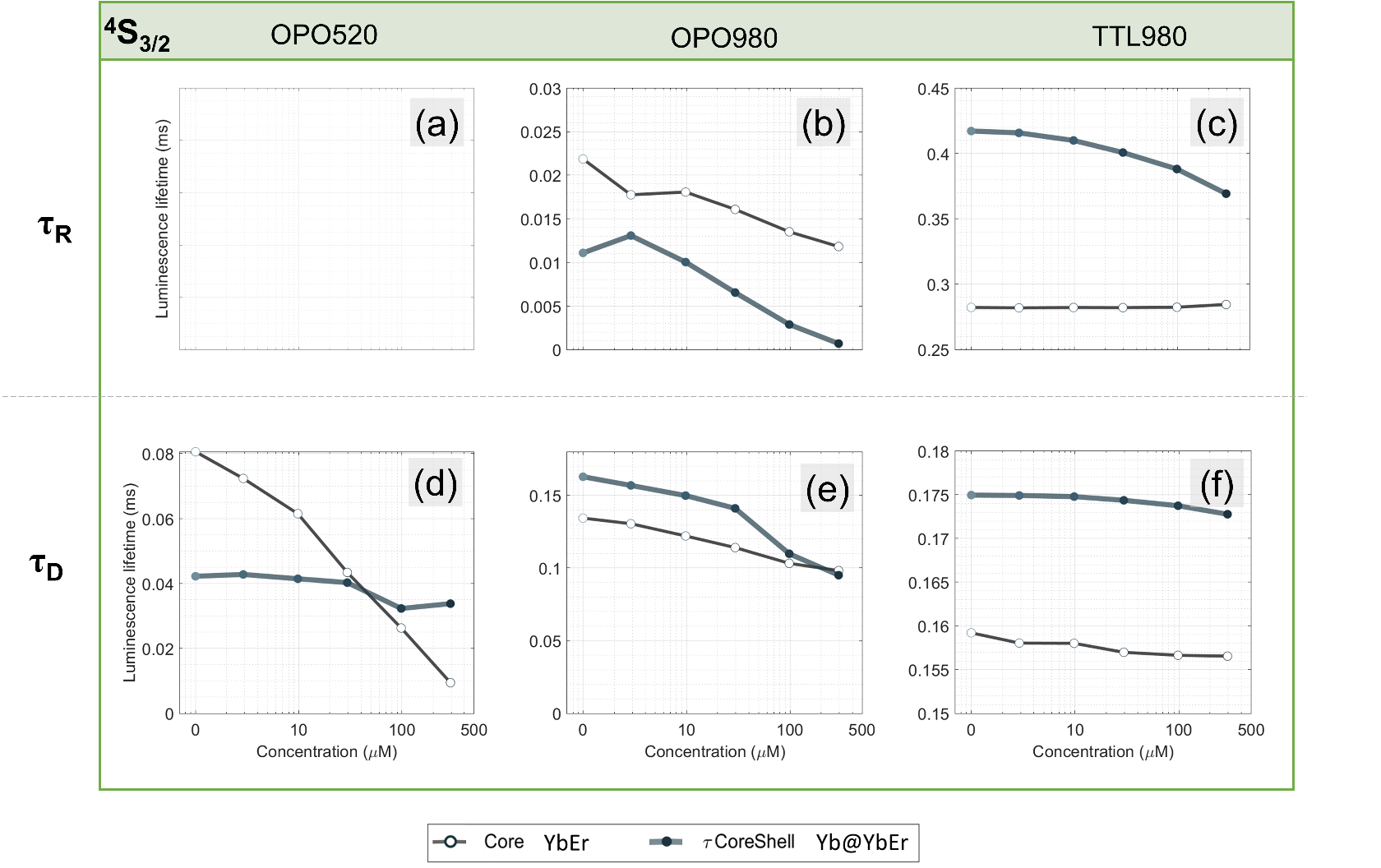


**Figure S8: Luminescence lifetimes for ^4^S_3/2_ energy level of Er^3+^ ions**. Luminescence lifetimes are split into different rows – rise time (b, c), decay averaged lifetime (d, e, f) for different photoexcitation mode: direct Er^3+^ ions photoexcitation with OPO520 (a, d), Yb^3+^ ions photoexcitation with OPO980 (b, e,), and Yb^3+^ ions photoexcitation with longer pulse TTL980 (c, fi). Dark grey with filled dots shows results for the core sample (NaYF_4_: 20% Yb^3+^ 2% Er^3+^), and light grey with empty circles shows results for the core-shell sample (NaYF_4_: 20% Yb^3+^ @ NaYF_4_: 20% Yb^3+^ 5% Er^3+^). No data are provided in (a) because no rise times were observed under Stokes excitation (at 520 nm).


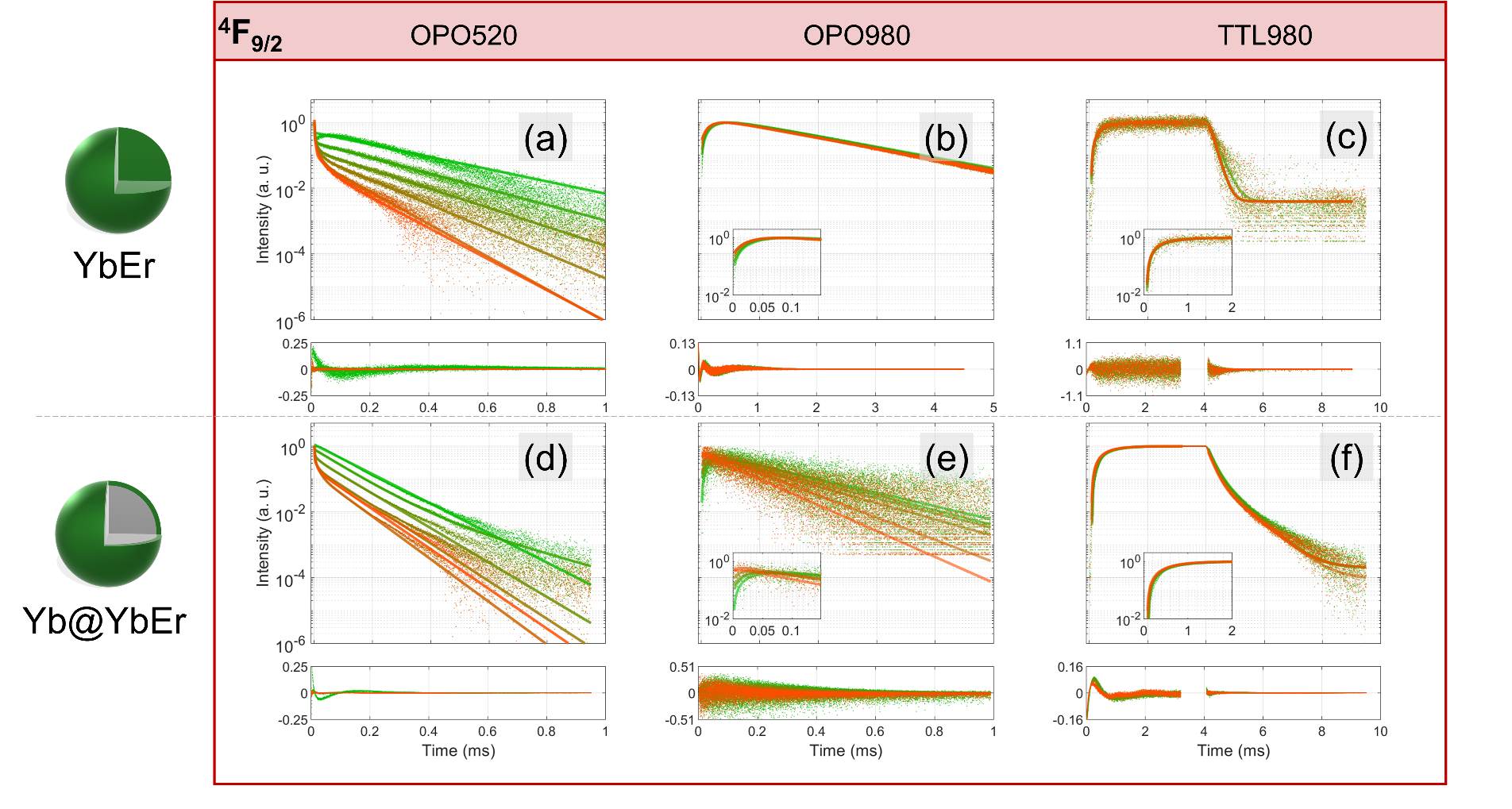


**Figure S9: Luminescence kinetics of** ^4^F_9/2_ energy level of Er^3+^ ions for different photoexcitation modes – direct Er^3+^ ions photoexcitation with OPO520 (a, d), Yb^3+^ ions photoexcitation with OPO980 (b, e), and Yb^3+^ ions photoexcitation with longer pulse TTL980 (c, f). First row (a, b, c) shows results for core sample (NaYF_4_: 20% Yb^3+^ 2% Er^3+^), 2^nd^ row (d, e, f) shows results for core-shell sample (NaYF_4_: 20% Yb^3+^ @ NaYF_4_: 20% Yb^3+^ 5% Er^3+^). The residual plots below luminescence lifetime.


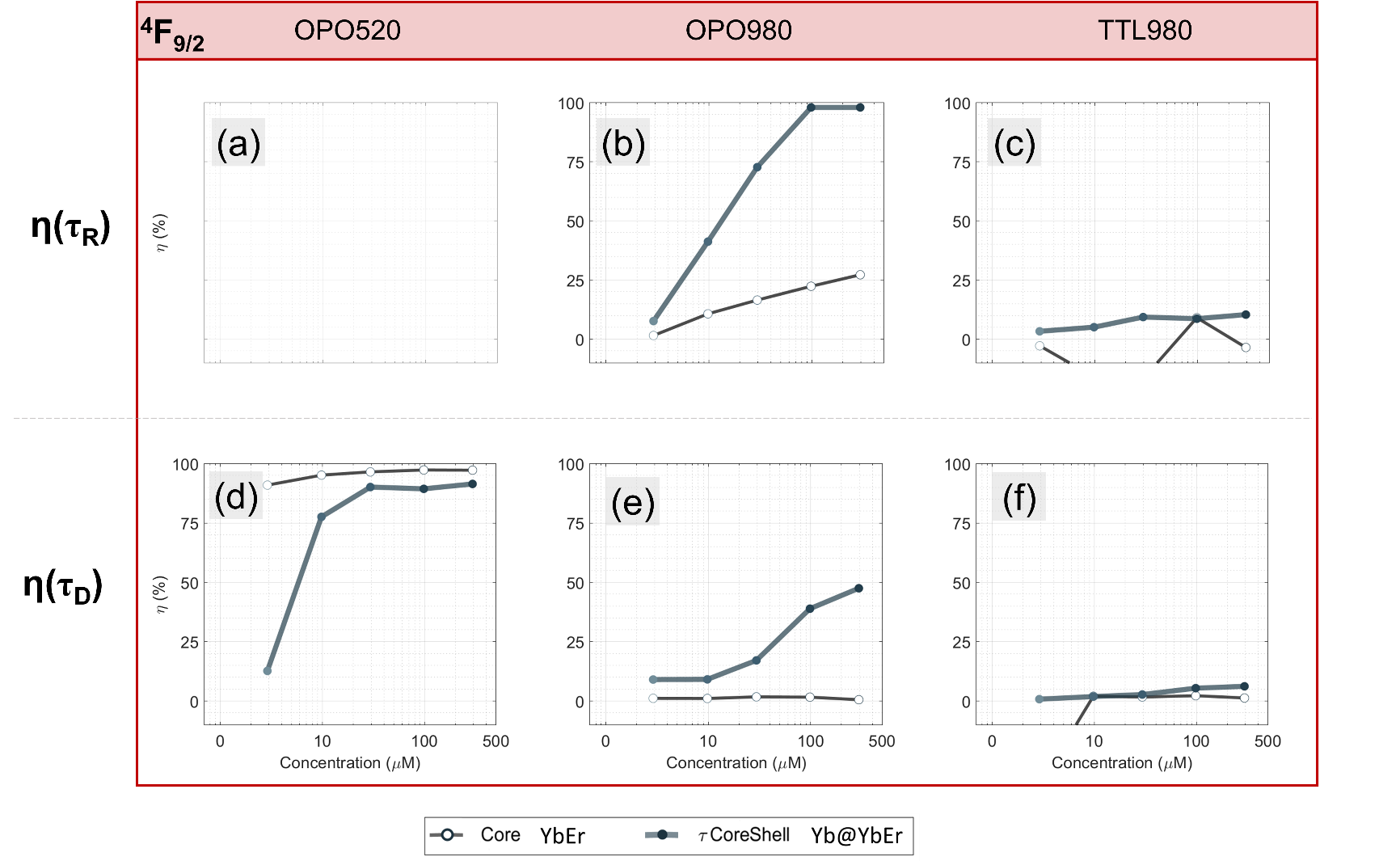


**Figure S10: LRET efficiencies based on luminescence kinetics from ^4^F_9/2_** energy level of Er^3+^ ions. Efficiencies are based on luminescence lifetimes of D with the absence of A versus D only lifetime, based on rise time (a, b, c), averaged lifetime (d, g, h) for different photoexcitation mode: direct Er^3+^ ions photoexcitation with OPO520 (a, d), Yb^3+^ ions photoexcitation with OPO980 (b, e), and Yb^3+^ ions photoexcitation with longer pulse TTL980 (c, f). Dark grey with filled dots shows results for the core sample (NaYF_4_: 20% Yb^3+^ 2% Er^3+^), and light grey with empty circles shows results for the core-shell sample (NaYF_4_: 20% Yb^3+^ @ NaYF_4_: 20% Yb^3+^ 5% Er^3+^). No data are provided in (a) because no rise times were analyzed under Stokes excitation (at 520 nm).


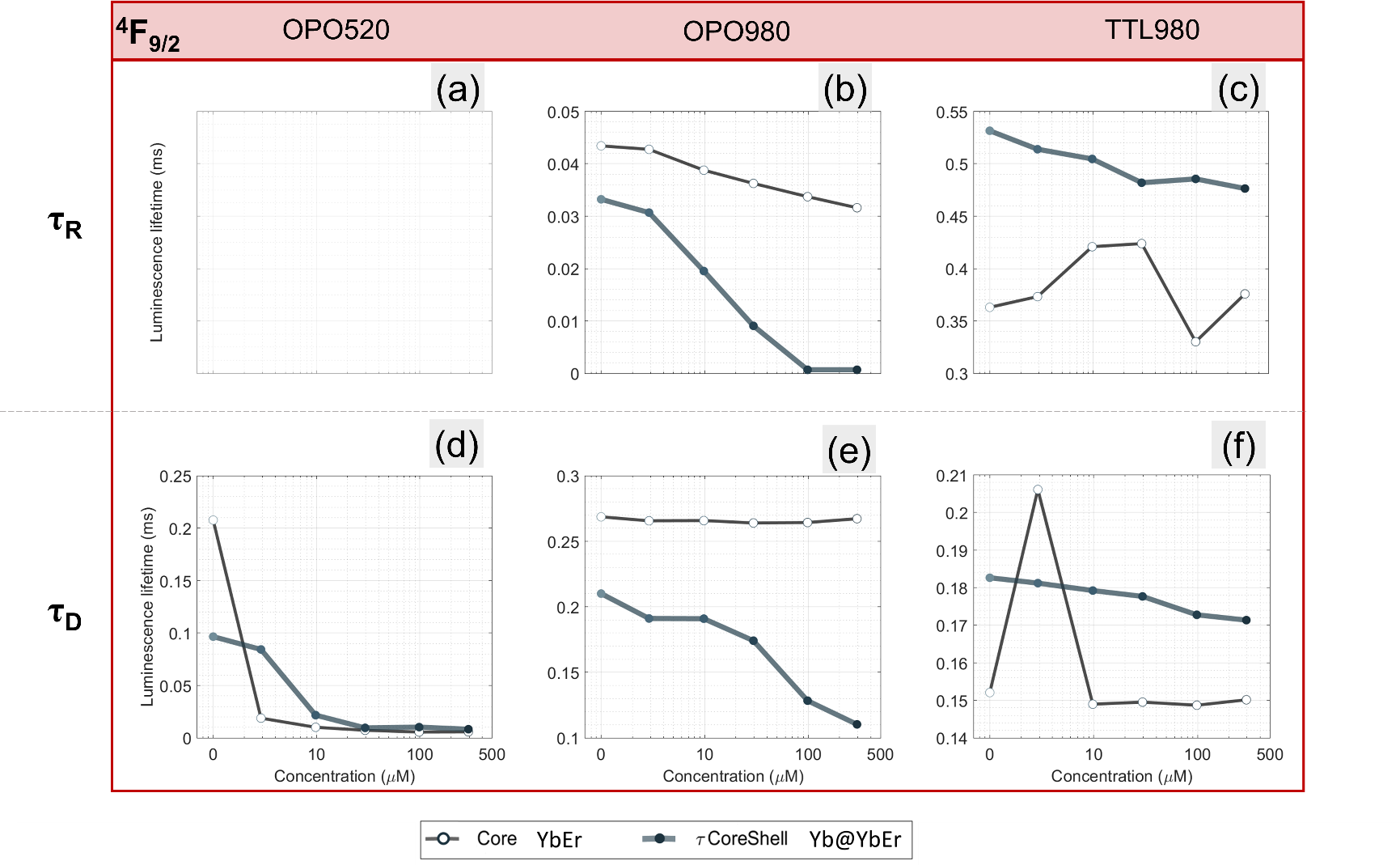


**Figure S11: Luminescence lifetimes for ^4^F_9/2_** energy level of Er^3+^ ions. Luminescence lifetimes are split into different rows – rise times (a, b, c) and averaged decay lifetime (d, e, f) for different photoexcitation mode: direct Er^3+^ ions photoexcitation with OPO520 (a, d), Yb^3+^ ions photoexcitation with OPO980 (b, e), and Yb^3+^ ions photoexcitation with longer pulse TTL980 (c, f). Dark grey with filled dots shows results for the core sample (NaYF_4_: 20% Yb^3+^ 2% Er^3+^), and light grey with empty circles shows results for the core-shell sample (NaYF_4_: 20% Yb^3+^ @ NaYF_4_: 20% Yb^3+^ 5% Er^3+^). No data are provided in (a) because no rise times were analyzed under Stokes excitation (at 520 nm).

**Table S3: Values of luminescence lifetimes** – rise and decay lifetimes for three photoexcitation type for ^4^S_3/2_ energy. Uncertainty standard error is calculated based on the residual error and number of points (n) $error =\frac{\sqrt{\frac{1}{n-1}}\sum residuals^{2}}{\sqrt{n}}$

| Energy level | | | τ ^4^S_3/2_ | | | | | | | | |
| --- | --- | --- | --- | --- | --- | --- | --- | --- | --- | --- | --- |
| Laser | | | OPO 520 | | | OPO980 | | | TTL980 | | |
| equation | | | triple exponential | | | differential rate equation | | | mono and double exponential | | |
| concentration | | | $\tau_{R}$ | $\tau_{\mathrm{Daverage}}$ | error | $\tau_{R}$ | $\tau_{D}$ | error | $\tau_{R}$ | $\tau_{D}$ | error |
|  |  | µM | μs | μs |  | μs | μs |  | μs | μs |  |
| Architecture | Core | 0 | - | 80.58 | 0.04 | 21.86 | 134.33 | 0.03 | 282.0 | 159.0 | 1.2 |
|  |  | 2.9 | - | 72.35 | 0.04 | 17.75 | 130.48 | 0.04 | 282.0 | 158.0 | 1.1 |
|  |  | 9.8 | - | 61.49 | 0.04 | 18.07 | 122.05 | 0.03 | 282.0 | 158.0 | 1.2 |
|  |  | 29.5 | - | 43.41 | 0.03 | 16.08 | 114.06 | 0.03 | 282.0 | 157.0 | 1.1 |
|  |  | 98.3 | - | 26.28 | 0.03 | 13.51 | 103.21 | 0.03 | 282.0 | 157.0 | 1.3 |
|  |  | 294.8 | - | 9.45 | 0.02 | 11.82 | 98.24 | 0.03 | 285.0 | 157.0 | 1.4 |
|  | Core Shell | 0 | - | 42.23 | 0.03 | 11.09 | 162.79 | 0.74 | 417.10 | 174.95 | 0.031 |
|  |  | 2.9 | - | 42.78 | 0.02 | 13.08 | 156.82 | 0.68 | 415.69 | 174.91 | 0.032 |
|  |  | 9.8 | - | 41.45 | 0.03 | 10.04 | 149.81 | 0.67 | 409.91 | 174.78 | 0.033 |
|  |  | 29.5 | - | 40.28 | 0.02 | 6.55 | 141.05 | 0.61 | 400.67 | 174.36 | 0.050 |
|  |  | 98.3 | - | 32.31 | 0.02 | 2.89 | 109.80 | 0.58 | 388.07 | 173.74 | 0.061 |
|  |  | 294.8 | - | 33.79 | 0.05 | 0.70 | 95.08 | 0.55 | 369.22 | 172.77 | 0.063 |

**Table S4: Values of luminescence lifetimes** – rise and decay lifetimes for three photoexcitation type ^4^F_9/2_ energy level. Uncertainty standard error is calculated based on the residual error and number of points (n) $error =\frac{\sqrt{\frac{1}{n-1}}\sum residuals^{2}}{\sqrt{n}}$

| Energy level | | | τ ^4^F_9/2_ | | | | | | | | |
| --- | --- | --- | --- | --- | --- | --- | --- | --- | --- | --- | --- |
| Laser | | | OPO 520 | | | OPO980 | | | TTL980 | | |
| equation | | | triple exponential | | | differential rate equation | | | mono and double exponential | | |
| concentration | | | $\tau_{R}$ | $\tau_{Daverage}$ | error | $\tau_{R}$ | $\tau_{D}$ | error | $\tau_{R}$ | $\tau_{D}$ | error |
|  |  | µM | μs | μs |  | μs | μs |  | μs | μs |  |
| Architecture | Core | 0 | - | 207.68 | 0.31 | 43.41 | 268.63 | 0.03 | 363.1 | 152.1 | 0.91 |
|  |  | 2.9 | - | 18.81 | 0.06 | 42.74 | 265.59 | 0.03 | 373.3 | 206.1 | 0.82 |
|  |  | 9.8 | - | 10.09 | 0.03 | 38.76 | 265.75 | 0.03 | 420.8 | 149.0 | 0.86 |
|  |  | 29.5 | - | 7.25 | 0.02 | 36.24 | 263.96 | 0.03 | 423.8 | 149.5 | 0.93 |
|  |  | 98.3 | - | 5.57 | 0.02 | 33.71 | 264.28 | 0.03 | 330.2 | 148.7 | 0.99 |
|  |  | 294.8 | - | 5.76 | 0.07 | 31.60 | 267.19 | 0.03 | 375.8 | 150.1 | 1.02 |
|  | Core Shell | 0 | - | 96.55 | 0.32 | 33.21 | 210.07 | 1.05 | 531.43 | 182.63 | 0.048 |
|  |  | 2.9 | - | 84.30 | 0.07 | 30.67 | 191.08 | 0.93 | 513.79 | 181.20 | 0.045 |
|  |  | 9.8 | - | 21.66 | 0.02 | 19.53 | 190.93 | 0.78 | 504.56 | 179.22 | 0.038 |
|  |  | 29.5 | - | 9.54 | 0.01 | 9.08 | 174.13 | 0.67 | 481.87 | 177.66 | 0.028 |
|  |  | 98.3 | - | 10.26 | 0.02 | 0.70 | 128.37 | 0.64 | 485.51 | 172.76 | 0.026 |
|  |  | 294.8 | - | 8.27 | 0.02 | 0.70 | 110.30 | 0.61 | 476.32 | 171.35 | 0.028 |

**Table S5: Values of RET efficiencies** based on luminescence lifetimes – the rise and decay lifetimes for three photoexcitation types for ^4^S_3/2_ energy level.

| Energy level | | | η ^4^S_3/2_ | | | | | |
| --- | --- | --- | --- | --- | --- | --- | --- | --- |
| Laser | | | OPO 520 | | OPO980 | | TTL980 | |
| equation | | | triple exponential | | differential rate equation | | mono and double exponential | |
| concentration | | | $\eta_{R}$ | $\eta_{Daverage}$ | $\eta_{R}$ | $\eta_{D}$ | $\eta_{R}$ | $\eta_{D}$ |
|  |  | µM | % | % | % | % | % | % |
| Architecture | Core | 0 | - | - | - | - | - | - |
|  |  | 2.9 | - | 10.21 | 18.78 | 2.86 | 1.38 | 4.90 |
|  |  | 9.8 | - | 23.69 | 17.33 | 9.14 | 15.14 | 9.52 |
|  |  | 29.5 | - | 46.13 | 26.43 | 15.09 | 24.97 | 15.35 |
|  |  | 98.3 | - | 67.38 | 38.18 | 23.17 | 36.88 | 23.21 |
|  |  | 294.8 | - | 88.27 | 45.92 | 26.87 | 42.90 | 27.25 |
|  | Core Shell | 0 | - | - | - |  | - | - |
|  |  | 2.9 | - | -1,.30 | -17.92 | 3.66 | 0.34 | 0.03 |
|  |  | 9.8 | - | 1.85 | 9.49 | 7.97 | 1.72 | 0.10 |
|  |  | 29.5 | - | 4.62 | 40.96 | 13.35 | 3.94 | 0.34 |
|  |  | 98.3 | - | 23.50 | 73.97 | 32.55 | 6.96 | 0.69 |
|  |  | 294.8 | - | 20.00 | 93.69 | 41.59 | 11.48 | 1.24941 |

**Table S6: Values of RET efficiencies** based on luminescence lifetimes – the rise and decay lifetimes for three photoexcitation types for ^4^F_9/2_ energy level.

| Energy level | | | η ^4^F_9/2_ | | | | | |
| --- | --- | --- | --- | --- | --- | --- | --- | --- |
| Laser | | | OPO 520 | | OPO980 | | TTL980 | |
| Equation | | | triple exponential | | differential rate equation | | mono exponential | |
| Concentration | | | $\eta_{R}$ | $\eta_{Daverage}$ | $\eta_{R}$ | $\eta_{D}$ | $\eta_{R}$ | $\eta_{D}$ |
|  |  | µM | % | % | % | % | % | % |
| Architecture | Core | 0 | - | - | - | - | - | - |
|  |  | 2.9 | - | 90.94 | 1.54 | 1.13 | -2.81 | 35.54 |
|  |  | 9.8 | - | 95.14 | 10.69 | 1.07 | -15.91 | 2.04 |
|  |  | 29.5 | - | 96.51 | 16.51 | 1.74 | -16.74 | 1.68 |
|  |  | 98.3 | - | 97.32 | 22.34 | 1.62 | 9.04 | 2.22 |
|  |  | 294.8 | - | 97.23 | 27.20 | 0.53 | -3.52 | 1.27 |
|  | Core Shell | 0 | - | - | - | - | - | - |
|  |  | 2.9 | - | 12.68 | 7.64 | 9.04 | 3.32 | 0.78 |
|  |  | 9.8 | - | 77.56 | 41.21 | 9.11 | 5.06 | 1.87 |
|  |  | 29.5 | - | 90.12 | 72.67 | 17.11 | 9.33 | 2.72 |
|  |  | 98.3 | - | 89.37 | 97.89 | 38.89 | 8.64 | 5.40 |
|  |  | 294.8 | - | 91.44 | 97.89 | 47.49 | 10.37 | 6.18 |

# Mathematical methods (kinetic modelling)

The luminescence lifetime model was implemented with differential rate equations (DRE) (**Table S9**) for transitions between energy levels inside Yb^3+^ and Er^3+^ ions, and Rose Bengal dye, and the results are presented in **Figures 5**, **S14**, and **S15**. Time-dependent changes of energy transitions between ions are mainly dependent on the distance between Er^3+^ ions and RB. However additional parameters such as concentration of *D* and *A*, photoexcitation flux, or inert transition can provide a significant role for overall RET efficiency value. In model analogically to the provided experiments, we simulate three types of photoexcitation pulse, with two based on indirect photoexcitation through Yb^3+^ ions with wavelength 980 nm – long (TTL980) or short (OPO980), and one based on direct photoexcitation of Er^3+^ ions with short pulses with wavelength 520 nm (OPO520).

Model of DRE proposed by Anderson et. al ^12^ new model with corrected parameters energy transfer values is well fitted to pulse laser measurements. The standard model corresponds better to long pulse measurement. That can be explained with improved measurements setup for lanthanide ions, and nowadays possibility of using the shorter photoexcitation pulses for lifetime curve analysis. Probably with very short pulses with higher energy, there are activated different processes that are more visible on the experimental and theoretical values. That leads to the conclusion that both models work very well with different energy regimes and time of pulse, but this leads us to check additional possibilities of energy scheme that corresponds to all tested photoexcitation models (experimentally and theoretically).

With an increasing concentration of RB acceptor dye, we can see faster rise and decay luminescence lifetimes for *D* energy level (^4^S_3/2_, Er^3+^), quenching of this level by LRET mechanism to RB attached, and should not be explained with blocking quenching to environment such as water (see material paragraph about Rose Bengal dye titration). Here we can also observe enhanced of red emission changes (^4^F_9/2_), which is not directly responsible for RET mechanism (due to energy overlap between from ^4^S_3/2_ energy level of Er^3+^ and RB acceptor dye). That could be explained by processes such as nonradiative emission from ^4^S_3/2_ to ^4^F_9/2_, energy migration, photoexcitation pathways, or Er^3+^, which influence the fulfillment of the energy level that is responsible for the LRET mechanism. For three different types of photoexcitation schemes, the preferable one with the highest efficiencies is for short and indirect photoexcitation pulses. Additionally, the concentration of ions or organic dyes involved in energy transfer processes and the value of W_ETU_ have a high impact on the explored processes, especially on the LRET changes visible in the rise and decay times of luminescence.

Comparing results from the model and trends from the experimental data, we assume that there is a very thin border between various types of excitation schemes. The first standard photoexcitation with 980 nm laser of Yb^3+^ ions, causes transition between following energy levels ^2^F_7/2_ → ^2^F_5/2_, afterward energy transfer up-conversion to Er^3+^ ions occurs. However, energy transfer up-conversion, energy migration, and double (to ^4^I_11/2_ energy level) or triple photoexcitation pathway (to ^4^G_11/2_ energy level) could have a significant role, which we observe in differential rate equations model and experiments (applying short and long time of photoexcitation laser). The second less typical proposed photoexcitation pathway is direct of Er^3+^ ions (between ^4^I_11/2_ → ^4^F_7/2_ energy levels, then we can observe nonradiative emission to the most interesting in a few of RET energy levels - ^4^S_3/2_ and ^2^H_11/2_), which is schematically shown in **Figure 1** and **Figure S12**.

## Equations and parameters for kinetics modeling

Implementation and solving differential rate equations for energy transitions levels of Yb and Er system (rate equation presented in **Table S9** and values of parameters presented in **Table S10**) are based on results of Bergstrand et al ^13^ and Anderson et al ^12^. The results are shown in **Figure 5**. Implementation of the differential equation were written in Matlab software, for solving differential equations, we used ode45 (based on the Runge-Kutta formula). The simplified energy level for an implemented model scheme of Yb^3+^ and Er^3+^ is presented in **Figures 1** and **S12**.


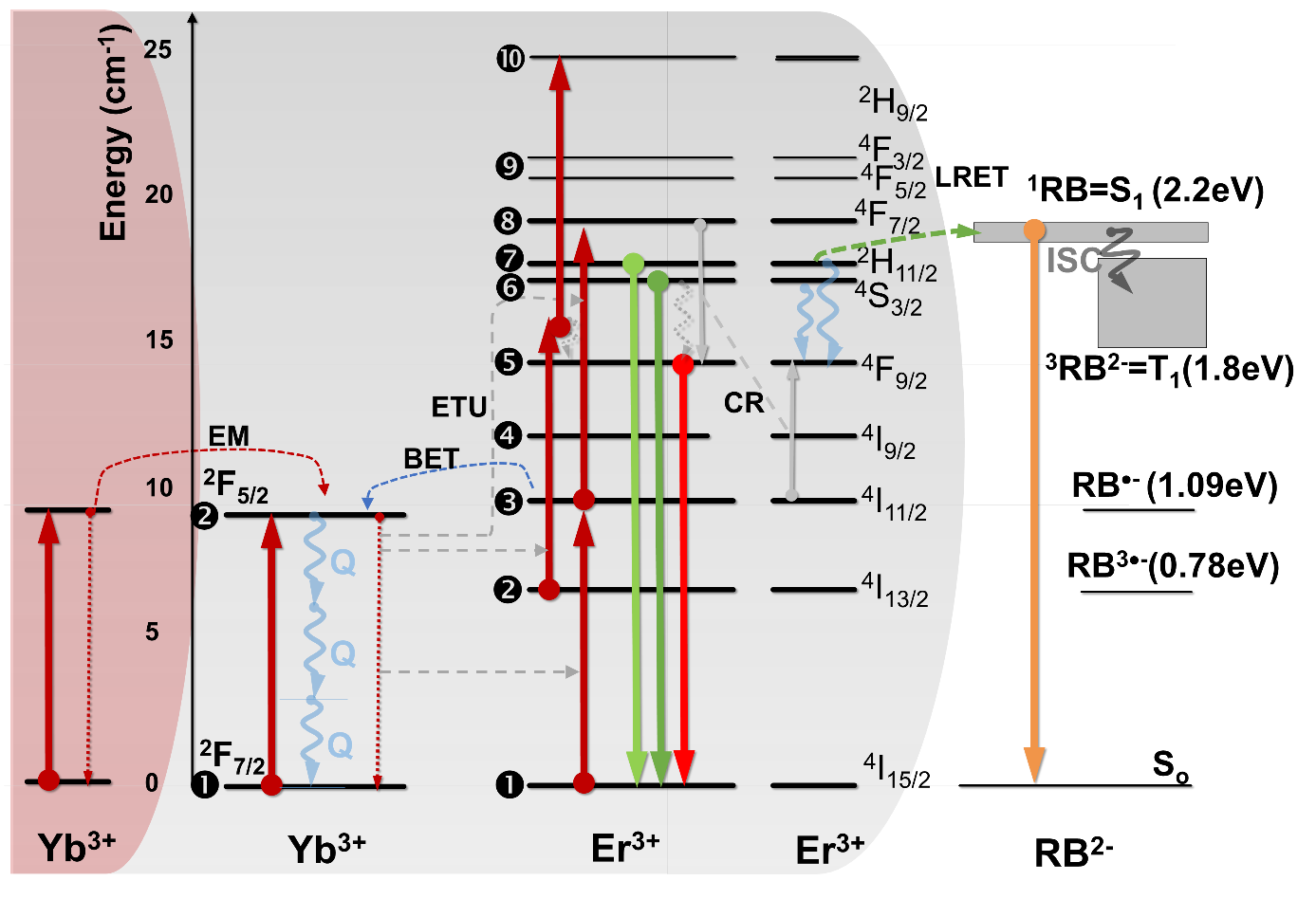


Figure S12: Simplified energy scheme of YbErRB system.

Astonishingly, the same rates were however, unsuitable for fitting the luminescence kinetics profiles for longer (4 ms) and low peak energy laser pulses from TTL controlled CW laser diode. In the latter case, the other set of energy transfer up-conversion rates gave better agreement. Actually, we found it striking that also previous literature reports on this Yb-Er system propose various DRE rate constants depending on the photoexcitation scheme. According to Berry *et al.*, despite the Yb-Er system being the most well known and most versatilely studied up-conversion system so far, there is still a need to revisit these parameters ^12^. Unfortunately, it seems impossible to obtain a general and exact set of DRE rate parameters, describing photoexcitation schemes well different. In particular, the same set of rate constant does not, currently allow to consider the (a) photoexcitation pulse length or photon flux (e.g. to compare short pulse (around 10 ns) to longer pulses (about a few milliseconds) excitations with high or low pulse energy). Moreover, this DRE model dismisses energy migration with Yb^3+^ sensitizer ^14^ or Er^3+^ ions ^15^ network between pairs of ions such as Yb – Er^14,16^ or Er – Tm ^17^ which have significant contribution quantified with simulations ^15,16,18^ in the donor luminescence and kinetics of the UC-LRET system. This means, that despite being relatively detailed, the existing Yb-Er DRE models are still incomplete.

Because the Yb^3+^ – Er^3+^ up-converting system is quite complex in terms of a number of interdependent processes, we have tried to adopt reasonable simplifications. First of all, we have considered the processes occurring only between energy levels below ^4^F_7/2_ level (with energy < 25000 cm^-1^) which anyway enables to comprehend the role of different modes of the photoexcitation on the up-conversion emission. Secondly, this model was further expanded with a singlet-singlet transition of the energy acceptor, whose excitation band spectrally overlaps with ^4^S_3/2_ + ^2^H_11/2_ energy levels of Er^3+^ donors. To do so, we have combined the above-mentioned DRE model with the *D-A* interaction term (explained in **SI** section **VI** Mathematical methods), which described how the acceptor modifies the kinetic behavior of major Er^3+^ energy levels (^4^S_3/2_ and ^4^F_9/2_). We could, therefore numerically solve these DRE to theoretically predict and gain some basic understanding on the way the RET phenomenon changes the temporal behavior of the Yb^3+^ - Er^3+^ up-converting system under different photoexcitation schemes. We performed our calculations for fixed parameters of pump power, energy transfer rates (tabulated in **Table S8**), and concentration of lanthanide ion dopants while testing the outcome of DRE modelling with just a few concentrations of Rose Bengal acceptor.

**Table S7: Rate equations** for YbEr system with RB organic dye used to model differential rate equations. I is the flux of photoexcitation laser energy, σ - absorption cross-section of lanthanide ions, n – fill of energy level, β – a fraction of ions that take the process in the energy transfer upconversion. The energy levels numbering used in the model is presented in the left column and **figure S12**.

| Energy level | | | Equation |
| --- | --- | --- | --- |
| $Yb_{1}$ | ^2^F_7/2_ | $\frac{\partial n_{Yb1}}{\partial t}=$ | $-I\cdot\sigma_{Yb}\cdot n_{Yb1}+n_{Yb2}\cdot k_{Yb}+n_{Yb2}\cdot k_{Ybrad}+k_{ET13}\cdot n_{Yb2}\cdot\beta_{ET}\cdot n_{Er1}+k_{ET25}\cdot n_{Yb2}\cdot\beta_{ET}\cdot n_{Er2}+k_{ET38}\cdot n_{Yb2}\cdot\beta_{ET}\cdot n_{Er3}+k_{ET59}\cdot n_{Yb2}\cdot\beta_{ET}\cdot n_{Er5}+k_{ET710}\cdot n_{Yb2}\cdot\beta_{ET}\cdot n_{Er7}-k_{ET31}\cdot n_{Yb1}\cdot\beta_{ET}\cdot n_{Er3}-k_{ET52}\cdot n_{Yb1}\cdot\beta_{ET}\cdot n_{Er5}-k_{ET83}\cdot n_{Yb1}\cdot\beta_{ET}\cdot n_{Er8}-k_{ET105}\cdot n_{Yb1}\cdot\beta_{ET}\cdot n_{Er10}$ |
| $Yb_{2}$ | ^2^F_5/2_ | $\frac{\partial n_{Yb\_2}}{\partial t} =$ | $- \frac{\partial n_{Yb1}}{\partial t}$ |
| $Er_{1}$ | ^4^I_15/2_ | $\frac{\partial n_{Er1}}{\partial t}$ | $-I\cdot\sigma_{Er}\cdot n_{Er1}-k_{ET13}\cdot n_{Yb2}\cdot\beta_{ET}\cdot n_{Er1}+k_{ET31}\cdot n_{Yb1}\cdot\beta_{ET}\cdot n_{Er3}-CR_{6}\cdot n_{Er1}\cdot n_{Er7}-CR_{4}\cdot n_{Er1}\cdot n_{Er4}+k_{UC2}\cdot n_{Er2}\cdot n_{Er2}+k_{R21}\cdot n_{Er2}+0.81\cdot k_{R31}\cdot n_{Er3}+0.9\cdot n_{Er5}\cdot k_{R51}+0.7\cdot n_{Er7}\cdot k_{R71}+0.4\cdot k_{R91}\cdot n_{Er9}+k_{FRET1}\cdot n_{Er7}\cdot n_{AccGr}+k_{FRET2}\cdot n_{Er5}\cdot n_{AccGr}$ |
| $Er_{2}$ | ^4^I_13/2_ | $\frac{\partial n_{Er2}}{\partial t}=$ | $-k_{ET25}\cdot n_{Yb2}\cdot\beta_{ET}\cdot n_{Er2}+k_{ET52}\cdot n_{Yb1}\cdot\beta_{ET}\cdot n_{Er5}+CR_{6}\cdot n_{Er1}\cdot n_{Er7}+2\cdot CR_{4}\cdot n_{Er1}\cdot n_{Er4}-2\cdot k_{UC2}\cdot n_{Er2}\cdot n_{Er2}-k_{R21}\cdot n_{Er2}+k_{NR3}\cdot n_{Er3}+0.19\cdot k_{R31}\cdot n_{Er3}+0.05\cdot n_{Er5}\cdot k_{R51}+0.25\cdot n_{Er7}\cdot k_{R71}+0.42\cdot k_{R91}\cdot n_{Er9}$ |
| $Er_{3}$ | ^4^I_11/2_ | $\frac{\partial n_{Er3}}{\partial t}=$ | $k_{ET13}\cdot n_{Yb2}\cdot\beta_{ET}\cdot n_{Er1}-k_{ET31}\cdot n_{Yb1}\cdot\beta_{ET}\cdot n_{Er3}-k_{ET38}\cdot n_{Yb2}\cdot\beta_{ET}\cdot n_{Er3}+k_{ET83}\cdot n_{Yb1}\cdot\beta_{ET}\cdot n_{Er8}+CR_{6}\cdot n_{Er1}\cdot n_{Er7}-k_{NR3}\cdot n_{Er3}+k_{NR4}\cdot n_{Er4}-k_{R31}\cdot n_{Er3}+0.05\cdot n_{Er5}\cdot k_{R51}+0.05\cdot n_{Er7}\cdot k_{R71}+0.14\cdot k_{R91}\cdot n_{Er9}$ |
| $Er_{4}$ | ^4^I_9/2_ | $\frac{\partial n_{Er4}}{\partial t}=$ | $k_{NR5}\cdot n_{Er5}-k_{NR4}\cdot n_{Er4}-CR_{4}\cdot n_{Er1}\cdot n_{Er4}+k_{UC2}\cdot n_{Er2}\cdot n_{Er2}$ |
| $Er_{5}$ | ^4^F_9/2_ | $\frac{\partial n_{Er5}}{\partial t}=$ | $k_{ET25}\cdot n_{Yb2}\cdot\beta_{ET}\cdot n_{Er2}-k_{ET52}\cdot n_{Yb1}\cdot\beta_{ET}\cdot n_{Er5}-k_{ET59}\cdot n_{Yb2}\cdot\beta_{ET}\cdot n_{Er5}+k_{ET105}\cdot n_{Yb1}\cdot\beta_{ET} \cdot n_{Er10}-n_{Er5}\cdot k_{R51}-k_{NR5}\cdot n_{Er5}+k_{NR7}\cdot n_{Er7}+0.04\cdot k_{R91}\cdot n_{Er9}-k_{FRET2}\cdot n_{Er5}\cdot n_{AccGr}$ |
| $Er_{6}$ | ^4^S_3/2_ | $\frac{\partial n_{Er6}}{\partial t}=$ | $0.0000$ |
| $Er_{7}$ | (^4^S_3/2_, ^2^H_11/2_) (real ^2^H_11/2_) | $\frac{\partial n_{Er7}}{\partial t}=$ | $-k_{ET710}\cdot n_{Yb2}\cdot\beta_{ET}\cdot n_{Er7}+I\cdot\sigma_{Er}\cdot n_{Er1}-CR_{6}\cdot n_{Er1}\cdot n_{Er7}-n_{Er7}\cdot k_{R71}-k_{NR7}\cdot n_{Er7}+k_{NR8}\cdot n_{Er8}-k_{FRET1}\cdot n_{Er7}\cdot n_{AccGr}$ |
| $Er_{8}$ | ^4^F_7/2_ | $\frac{\partial n_{Er8}}{\partial t}=$ | $k_{ET38}\cdot n_{Yb2}\cdot\beta_{ET}\cdot n_{Er3}-k_{ET83}\cdot n_{Yb1}\cdot\beta_{ET}\cdot n_{Er8}+k_{NR9}\cdot n_{Er9}-k_{NR8}\cdot n_{Er8}$ |
| $Er_{9}$ | ^4^F_5/2_, ^4^F_3/2_ | $\frac{\partial n_{Er9}}{\partial t}=$ | $k_{ET59}\cdot n_{Yb2}\cdot\beta_{ET}\cdot n_{Er5}-k_{NR9}\cdot n_{Er9}+k_{NR10}\cdot n_{Er10}-k_{R91}\cdot n_{Er9}$ |
| $Er_{10}$ | ^2^H_9/2_ | $\frac{\partial n_{Er10}}{\partial t}=$ | $k_{ET710}\cdot n_{Yb2}\cdot\beta_{ET}\cdot n_{Er7}-k_{ET105}\cdot n_{Er10}\cdot n_{Yb1}\cdot\beta_{ET}-k_{NR10}\cdot n_{Er10}$ |
| RB | RB_0_ | $\frac{\partial n_{RB_{0}}}{\partial t}=$ | $-k_{FRET1}\cdot n_{Er7}\cdot n_{RB_{0}}+k_{Sing}\cdot n_{RB_{Sing}}$ |
| RB | RB_singlet_ | $\frac{\partial n_{RB_{\mathrm{singlet}}}}{\partial t}=$ | $k_{FRET1}\cdot n_{Er7}\cdot n_{RB_{0}}- k_{Sing}\cdot n_{RB_{Sing}}$ |

Most of the data due to the same physical constant are easily comparable between experiment and theoretical model (such as photoexcitation wavelength, time, or energy of pulse). Because of the unification of physical constants between multiplied values, the concentration of dopant ion in the given volume in theoretical calculations are given in cm^-3^ (in which is also given energy levels of Ln^3+^ values and values of transition probabilities rates), while in experiment concentration are given in µM (due to the calculations of volume and mass of used constants).

Power density, energy and flux of energy used in model for photoexcitation wavelength from laser is presented in equation below ^19^

| $Power Density=\frac{Power}{S}=\frac{Power}{\pi\cdot r^{2}} = \frac{power}{\pi\cdot\left( 1.8 \cdot\sigma_{Laser} \right)^{2}}$ | Eq. S10 |
| --- | --- |

Two physical parameters that ate included in Planck constant $h=6.62607004\cdot{10}^{-34}\left[ m^{2}\cdot kg{\cdot s}^{-1} \right]$, speed of light $c = 299792458 \left[ m\cdot s^{-1} \right]$

| $Power Density=\frac{Power}{S}=\frac{Power}{\pi\cdot r^{2}} = \frac{Power}{\pi\cdot\left( 1.8 \cdot\sigma_{Laser} \right)^{2}}$ | Eq. S11 |
| --- | --- |
| $Energy =\frac{h \cdot c}{\lambda}$ | Eq. S12 |
| $Flux =\frac{Power Density}{Energy}\left[ \frac{m^{2}}{s} \right]$ | Eq. S13 |

The acceptor (RB) concentration were provided for calculations as RB molecules averaged over ions per cm^-3^, taking into account normalized k_LRET_ between *D*-*A* pair and assuming a number of RB in the following numbers: 0, 10^19^, 10^20^, 10^21^, 10^22^, 10^23^, 10^24^. Assuming that W_RET_ includes a number of acceptors (N­A) and critical concentration, which is dependent on *D*– *A* distance, this parameter can be described as a micro parameter, and averaged for whole NPs for inside and outside Ln^3+^ ion dopant (with different values of R_DA_)

The concentration conversion can be described with equation ^20^

| $c =((n / (6.02\cdot{10}^{23})) mol)\cdot((1000 cm^{3}/L) / (V cm^{3}))$ | Eq. S14 |
| --- | --- |
| $c = 1000 \cdot n / (6.02\cdot{10}^{23} \cdot V) [mol / L]$ | Eq. S15 |

Results given in **figure** 5, were calculated with concentration in cm^-3^ but there were calculated and given in µMol – are respectively 0, 10^19^, 10^20^, 10^21^, 10^22^, 10^23^, 10^24^ (ions cm^-3^) are equivalent to 0, 0.0166, 0.1661, 1.6611, 16.6113, 166.1129, 1661.1296 (μM).

To determine macromolecular parameter how many about numbers of molecules of RB dye are per ion donor (Er^3+^ ions) for volume (cm^-3^) we fitted Inokuti-Hirayama model

| $I(t)=I(0)\cdot exp\left[ -\frac{t}{\tau_{D}}-\Gamma\left( 1-\frac{3}{m} \right)\frac{N_{A}}{N_{C}}\left( \frac{t}{\tau_{D}} \right)^{\frac{3}{m}} \right]$ | Eq. S16 |
| --- | --- |

where for dipole-dipole interaction $m=6$ and critical transfer concentration is equal to $Nc=3/(4\pi R_{DA}^{3})$ and we assume averaged in a range around 10^-21^ molecules cm^-3^) ^21,22^. Although we check how changes of RB concentration influence Er^3+^ emission (**figure 2**) for concentrations 0, 10^19^, 10^20^, 10^21^, 10^22^, 10^23^, 10^24^ (ions cm^-3^).

**Table S8: Table with values** of parameters taken to rate equation model with reference to the article or indicated that value is taken analogically to experimental condition or this is for calculations accuracy (as tolerance of calculation 10^-2^ and experimental data).

| Type of pulse | | TTL980 | OPO980 | OPO520 |  |
| --- | --- | --- | --- | --- | --- |
| parameter | Unit | value | | | Ref |
| λ | (nm) | $980$ | $980$ | $520$ | Experiment |
| Start time |  | $0$ | | | Experiment |
| Time Laser Pulse on | (ms) | $0.1$ | | | Experiment |
| Impulse Time |  | $4$ ms | $5-7$ ns | | Experiment |
| Time Cycle | (ms) | $10$ | $(1/20)\cdot\left( {10}^{3} \right)$ | | Experiment |
| Step of time | (ms) | $0.005$ | $0.001$ | | Experiment |
| Time end | (ms) | $10$ | | | Experiment |
| AbsTol |  | ${10}^{-2}$ | | | Calculations |
| RelTol |  | ${10}^{-4}$ | | | Calculations |
| Laser spot size | (µm^2^) | 0.0074 | | | ^19^ |
| Power | (mW) | $0.0093$ | $0.0028$ | 0.0042 | Calculated from energy |
| Power density | (mW cm^-2^) | $1.2539\cdot{10}^{6}$ | $3.8129\cdot{10}^{5}$ | $5.5996\cdot{10}^{5}$ | Calculated from energy |
| Energy | (mJ) | $0.093046729$ | $0.141466$ | $0.207752336$ | Experiment |
| Flux of energy | photons  s^-1^ cm^−2^ | 6.18626$\cdot{10}^{20}$ | 9.4054$\cdot{10}^{21}$ | 1.3812 $\cdot{10}^{21}$ | Calculated from energy |
| n_s-Yb_ | (ions cm^-3^) | $1.5\cdot{10}^{21}$ | | | ^13^ |
| n_a-Er_ | (ions cm^-3^) | $1.5\cdot{10}^{20} (or 1.37\cdot{10}^{20})$ | | | ^23^ |
| n_RB_ | (ions cm^-3^) | $\sim1.5\cdot{10}^{20}$ | | |  |
| σ_S_ | (cm^2^) | $1.5\cdot{10}^{-20}$ | | $0$ | ^13^ |
| σ_A_ | (cm^2^) | $0$ | | $1.5\cdot{10}^{-20}$ |  |
| k_ET13_ | (cm^3^ s^-1^) | $5.9\cdot2.2\cdot{10}^{-16}$ | | | ^12^ |
| k_ET25_ | (cm^3^ s^-1^) | $0$ | | | ^12^ |
| k_ET38_ | (cm^3^ s^-1^) | $1.54 \cdot{10}^{-15}$ | | | ^12^ |
| k_ET59_ | (cm^3^ s^-1^) | $1.76 \cdot{10}^{-15}$ | | | ^12^ |
| k_ET710_ | (cm^3^ s^-1^) | $6.07\cdot{10}^{-15}$ | | | ^12^ |
| k_ET31_ | (cm^3^ s^-1^) | $2\cdot{10}^{-16}$ | | | ^12^ |
| k_ET52_ | (cm^3^ s^-1^) | $0$ | | | ^12^ |
| k_ET83_ | (cm^3^ s^-1^) | $2.04 \cdot{10}^{-16}$ | | | ^12^ |
| k_ET105_ | (cm^3^ s^-1^) | $2.84 \cdot{10}^{-16}$ | | | ^12^ |
| C (cross-relaxation) | (cm^3^ s^-1^) | $2.5\cdot{10}^{-16}-2.5\cdot{10}^{-18}$ | | | ^13^ |
| CR_6_ | (cm^3^ s^-1^) | $2.79 \cdot{10}^{-17}$ | | | ^12^ |
| CR_4_ | (cm^3^ s^-1^) | $8.04 \cdot{10}^{-19}$ | | | ^12^ |
| k_UC2_ | (cm^3^ s^-1^) | $2.31\cdot{10}^{-17}$ | | | ^12^ |
| k_R21_ (1500 nm) | (s^-1^) | $110$ | | | ^12^ |
| k_R31_ (1 μm) | (s^-1^) | $73$ | | | ^12^ |
| k_R51_ (^4^F_9/2_ red) | (s^-1^) | $2039$ | | | ^12^ |
| k_R61_ (540 nm, ^4^S_3/2_ green) | (s^-1^) | $0$ | | | ^12^ |
| k_R71_ (520 nm, ^2^H_11/2_ green) | (s^-1^) | $1510$ | | | ^12^ |
| k_R91_ (410 nm, blue) | (s^-1^) | $2330$ | | | ^12^ |
| k_NR3_ | (s^-1^) | $61$ | | | ^12^ |
| k_NR4_ | (s^-1^) | $22120$ | | | ^12^ |
| k_NR5_ | (s^-1^) | $0$ | | | ^12^ |
| k_NR6_ | (s^-1^) | $0$ | | | ^12^ |
| k_NR7_ | (s^-1^) | $26$ | | | ^12^ |
| k_NR8_ | (s^-1^) | ${10}^{6}$ | | | ^12^ |
| k_NR9_ | (s^-1^) | $43450$ | | | ^12^ |
| k_NR10_ | (s^-1^) | $1.76\cdot{10}^{6}$ | | | ^12^ |
| R_0_ | (nm) | $5$ | | |  |
| r_DA_ | (nm) | $5$ | | |  |
| $k_{\mathrm{Singlet}}$ | (s^-1^) | $\frac{1}{\tau_{A}}=\frac{1}{\tau_{D}}$ | | |  |
| k_LRET1_ | (s^-1^) | $\tau_{D1}^{-1}\cdot\left( \frac{R_{0}}{r_{\mathrm{DA}}} \right)^{6}/n_{aEr}$ | | | ^2^ |
| k_R_ | (s^-1^) | $1/\tau_{R}$ | | |  |

## Influence of power and concentration on donor luminescence lifetimes

Energy transfer energy value can influence the luminescence curves of Er^3+^ ions. The tendency of luminescence decay curves changes of Er^3+^ ions as acceptor influence behavior and analysis of Resonance Energy Transfer between Er^3+^ and RB (**figure S13**). Power changes in theoretical measurements, show that maximal implemented power gives luminescence decay lifetimes without rise times, probably due to high pump power (**figure S14**). Starting parameters were analogical with values to the experiments. Although probably not 100 % pumping power in experiment gives energy to Yb^3+^ ions. This can be caused due to scattering of energy by cuvette or solution in which nanocrystals with dyes are dissolved. According to **Figures S13** and **S14** we can assume that a faster energy transfer rate (k_ET_) between Yb^3+^ and Er^3+^ ions and smaller than maximal refill of energy levels under photoexcitation power can cause donor luminescence lifetime curve to be more susceptible to changes under acceptor presence.

In the simulation (differential rate equations), we implemented three types of photoexcitation pulse, including proper length of pulse and value of energy analogical used in the experiment. In rate equations, we define concentrations and a fraction of ions involved in Energy Transfer Upconversion (ETU) between Yb^3+^ and Er^3+^ ions. As a proposal based on our experimental and theoretical data, there is observed that photoexcitation can emphasize different transitions between different energy levels. Luminescence lifetime properties can be influenced by photoexcitation pulse time and energy. These types of input changes can expose more energy transfer between sensitizer (Yb^3+^) and activator (Er^3+^).

Based on the model luminescence lifetime curve for the donor, we can calculate the efficiency of RET, which indicate further changes. We indicated processes such as how fast energy is transferred between lanthanides ions (**Figure S13**) and how many donor ions (Er^3+^) we photoexcite with laser (**Figure S14**).


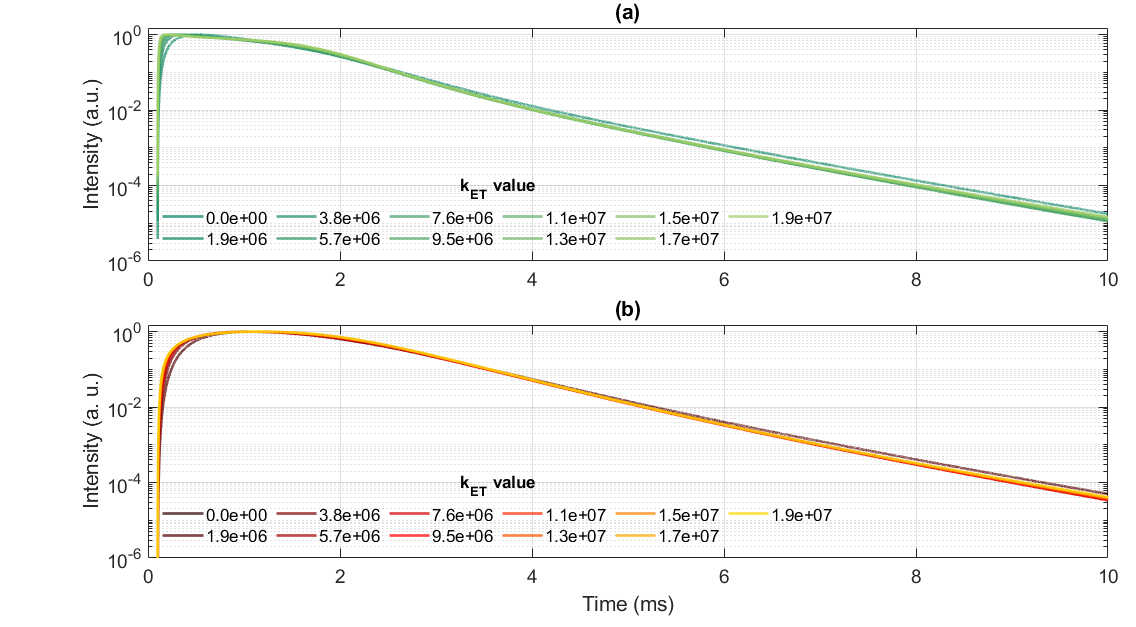


**Figure S13: The simulated luminescence lifetime curves for** emission from ^4^S_3/2_ and ^4^F­_9/2_ energy levels of Er^3+^, based on differential rate equations. Scan of changes for all ETU values between Yb^3+^ and Er^3+^ lanthanides ions, where value multiplier is included for each energy transfer rate k_ET_ (table S2, to resolve DRE presented in table S1) k_ET13_ = 5.9 ∙ 2.2 ∙ 10^-16^, k_ET25_ = 0, k_ET38_ = 1.54 ∙ 10^-15^, k_ET59_ = 1.76 ∙ 10^-15^, k_ET710_ = 6.07 ∙ 10^-15^, k_ET31_ = 2 ∙ 10^-16^, k_ET52_ = 0, k_ET83_ = 2.04 ∙ 10^-16^, k_ET105_ = 2.84 ∙ 10^-16^ (cm^3^ s^-1^).


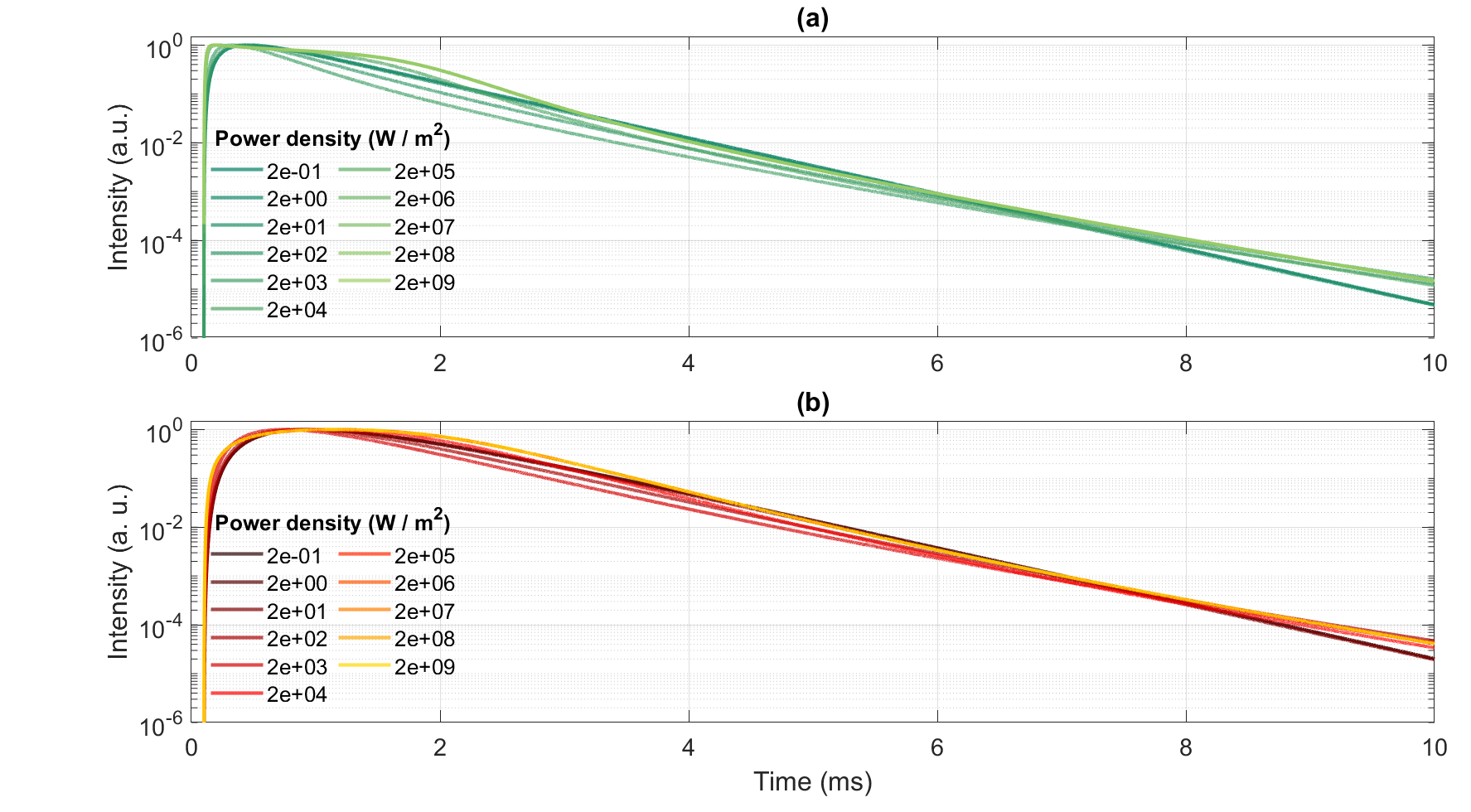


**Figure S14: The simulated luminescence lifetime curves for** emission from ^4^S_3/2_ and ^4^F_­9/2_ energy levels of Er^3+^ based on differential rate equations. Scan of values over photoexcitation power of Yb^3+^ ions, where values of power densities are equal $2\cdot{10}^{-1}$, $2\cdot{10}^{0}$, $2\cdot{10}^{1}$, $2\cdot{10}^{2}$, $2\cdot{10}^{3}$, $2\cdot{10}^{4}$, $2\cdot{10}^{5}$, $2\cdot{10}^{6}$, $2\cdot{10}^{7}$, $2\cdot{10}^{8}$, $2\cdot{10}^{9}$.

# References

1. Päkkilä, H. *et al*. Aptamer-Directed Lanthanide Chelate Self-Assembly for Rapid Thrombin Detection. *Analyst* **138**, 5107–5112. doi: 10.1039/C3AN00192J (2013).
2. Medintz, I. & Hildebrandt, N. FRET – Förster Resonance Energy Transfer. (Wiley, 2013).
3. Pilch‐Wrobel, A. *et al*. Engineering the compositional architecture of core‐shell upconverting lanthanide‐doped nanoparticles for optimal luminescent donor in resonance energy transfer: the effects of energy migration and storage. *Small* **18**, 2200464, doi: 10.1002/smll.202200464 (2022).
4. Kaminskii, A. A. Crystalline lasers Physical Processes and Operating Schemes. CRC Press, doi: 10.1109/ACCESS.2015.2424273 (1996).
5. Rose Bengal. In **Hawley’s Condensed Chemical Dictionary**, doi: 10.1002/9780470114735.hawley14117 (2007)
6. Algar, W. R. *et al*. Emerging Non-Traditional Förster Resonance Energy Transfer Configurations with Semiconductor Quantum Dots: Investigations and Applications. *Coordination Chemistry Reviews*, **263–264**, 65–85. doi: 10.1016/j.ccr.2013.07.015 (2014).
7. Marciniak, L. *et al*. Heterogeneously Nd^3+^ Doped Single Nanoparticles for NIR-Induced Heat Conversion, Luminescence, and Thermometry. *Nanoscale* **9**, 8288–8297. doi.org/10.1039/c7nr02630g (2017).
8. Tarai, M. *et al*. Inner Filter Effect and the Onset of Concentration Dependent Red Shift of Synchronous Fluorescence Spectra. *Analytica Chimica Acta* **940**, 113–119. doi: 10.1016/j.aca.2016.08.041 (2016).
9. Xu, D. *et al*. Aggregation of Rose Bengal Molecules in Solution. *Journal of Photochemistry and Photobiology A: Chemistry* **40**, 361–370. doi: 10.1016/1010-6030(87)85013-X (1987).
10. Mendes, B. *et al*. Influence of Rose Bengal Dimerization on Photosensitization. *Photochemistry and Photobiology* **97**, 718–726 doi: 10.1111/php.13379 (2021).
11. Nechaev, A. *et al*. The influence of energy migration on luminescence kinetics parameters in upconversion nanoparticles. *Nanotechnology* **28**, 035401. doi: 10.1088/1361-6528/28/3/035401 (2016).
12. Anderson, R. B. *et al*. Revisiting the NIR-to-Visible Upconversion Mechanism in β-NaYF_4_: Yb^3+^,Er^3+^. *The Journal of Physical Chemistry A* **5**, 36–42. doi.org/10.1021/jz402366r (2014).
13. Bergstrand, J. *et al*. On the Decay Time of Upconversion Luminescence. *Nanoscale* **11**, 4959–4969. doi: 10.1039/C8NR10332A (2019).
14. Teitelboim, A. *et al*. Energy Transfer Networks within Upconverting Nanoparticles Are Complex Systems with Collective, Robust, and History-Dependent Dynamics. *The Journal of Physical Chemistry C* **123**, 2678–2689. doi.org/10.1021/acs.jpcc.9b00161 (2019).
15. Khoptyar, D. *et al*. Homogeneous upconversion in Er-doped fibers under steady state excitation: analytical model and its Monte Carlo verification. *Journal of the Optical Society of America B* **22**, 582-590. doi: 10.1364/JOSAB.22.000582 (2005).
16. Grauel, B. *et al*. Volume and Surface Effects on Two-Photonic and Three-Photonic Processes in Dry Co-Doped Upconversion Nanocrystals. *Nano Research* **2021**, 1–12 doi: 10.1007/S12274-021-3727-Y (2021).
17. Chan, E. M. *et al*. Concentrating and Recycling Energy in Lanthanide Codopants for Efficient and Spectrally Pure Emission: The Case of NaYF_4_: Er^3+^/Tm^3+^ Upconverting Nanocrystals. *The Journal of Physical Chemistry B* **116**, 10561–10570, doi: 10.1021/jp302401j (2012).
18. Zuo, J. *et al*. Precisely Tailoring Upconversion Dynamics via Energy Migration in Core–Shell Nanostructures. *Angewandte Chemie International Edition* **57**, 3054–3058. doi: 10.1002/anie.201711606 (2018).
19. May, P. S. *et al*. Tutorial on the Acquisition, Analysis, and Interpretation of Upconversion Luminescence Data. *Methods and Applications in Fluorescence* **7**, 023001. doi: 10.1088/2050-6120/ab02c6 (2019).
20. Skoog, D. A. *et al*. Principles of Instrumental Analysis; (2018).
21. Agazzi, L. *et al*. Energy-Transfer-Upconversion Models, Their Applicability and Breakdown in the Presence of Spectroscopically Distinct Ion Classes: A Case Study in Amorphous Al_2_O_3_:Er^3+^. *The Journal of Physical Chemistry C* **117**. doi: 10.1021/jp4011839 (2013)
22. Carrasco, I. *et al*. Super-quadratic upconversion luminescence among lanthanide ions, *Optics Express*, **27**, 33217-33232, doi: 10.1364/OE.27.033217 (2019).
23. Baride, A. *et al*. Cross-relaxation from Er^3+^(^2^H_11/2_,^4^S_3/2_) and Er^3+^(^2^H_9/2_) in β-NaYF_4_:Yb,Er and implications for modeling upconversion dynamics. *The Journal of Physical Chemistry C* **124**, 2193-2201, doi: 10.1021/acs.jpcc.9b10163 (2020).
